# Functional, metabolic and transcriptional maturation of human pancreatic islets derived from stem cells

**DOI:** 10.1038/s41587-022-01219-z

**Published:** 2022-03-03

**Authors:** Diego Balboa, Tom Barsby, Väinö Lithovius, Jonna Saarimäki-Vire, Muhmmad Omar-Hmeadi, Oleg Dyachok, Hossam Montaser, Per-Eric Lund, Mingyu Yang, Hazem Ibrahim, Anna Näätänen, Vikash Chandra, Helena Vihinen, Eija Jokitalo, Jouni Kvist, Jarkko Ustinov, Anni I. Nieminen, Emilia Kuuluvainen, Ville Hietakangas, Pekka Katajisto, Joey Lau, Per-Ola Carlsson, Sebastian Barg, Anders Tengholm, Timo Otonkoski

**Affiliations:** 1 Stem Cells and Metabolism Research Program, Faculty of Medicine, University of Helsinki, Helsinki, Finland; 2 Bioinformatics and Genomics Program, Centre for Genomic Regulation (CRG), The Barcelona Institute of Science and Technology (BIST), Barcelona, Spain; 3 Centro de Investigación Biomédica en Red de Diabetes y Enfermedades Metabólicas Asociadas (CIBERDEM), Barcelona, Spain; 4 Department of Medical Cell Biology, Uppsala University, Uppsala, Sweden; 5 Electron Microscopy Unit, Institute of Biotechnology, University of Helsinki, Helsinki, Finland; 6 Metabolomics Unit, Institute for Molecular Medicine Finland, Helsinki, Finland; 7 Institute of Biotechnology, Helsinki Institute of Life Science, University of Helsinki, Helsinki, Finland; 8 Molecular and Integrative Bioscience Research Program, Faculty of Biological and Environmental Sciences, University of Helsinki, Helsinki, Finland; 9 Department of Cell and Molecular Biology, Karolinska Institutet, Stockholm, Sweden; 10 Children’s Hospital, Helsinki University Hospital and University of Helsinki, Helsinki, Finland

**Keywords:** Stem-cell research, Type 1 diabetes, Experimental models of disease, Stem-cell differentiation, Regenerative medicine

## Abstract

Transplantation of pancreatic islet cells derived from human pluripotent stem cells is a promising treatment for diabetes. Despite progress in the generation of stem-cell-derived islets (SC-islets), no detailed characterization of their functional properties has been conducted. Here, we generated functionally mature SC-islets using an optimized protocol and benchmarked them comprehensively against primary adult islets. Biphasic glucose-stimulated insulin secretion developed during in vitro maturation, associated with cytoarchitectural reorganization and the increasing presence of alpha cells. Electrophysiology, signaling and exocytosis of SC-islets were similar to those of adult islets. Glucose-responsive insulin secretion was achieved despite differences in glycolytic and mitochondrial glucose metabolism. Single-cell transcriptomics of SC-islets in vitro and throughout 6 months of engraftment in mice revealed a continuous maturation trajectory culminating in a transcriptional landscape closely resembling that of primary islets. Our thorough evaluation of SC-islet maturation highlights their advanced degree of functionality and supports their use in further efforts to understand and combat diabetes.

## Abstract (web summary)

Pancreatic islets derived from stem cells are benchmarked against primary cells.

## Main

The generation of functional pancreatic beta cells from human pluripotent stem cells (hPSCs) is a main goal of stem cell research, aiming to provide a renewable and consistent source of cells for the treatment of diabetes. Stem-cell-derived beta cells could solve the limitations of using cadaveric donor islets for transplantation and serve as a model system to understand the pathogenic mechanisms leading to various forms of diabetes^1^. Several studies have differentiated hPSCs to cell clusters that closely resemble primary islets (SC-islets) using multistage in vitro differentiation protocols that mimic the sequential inductive signals controlling pancreatic islet development in vivo^2–9^. Individual studies have reported particular transcriptomic^7,10^, functional^3,8,9,11^ and metabolic^2,12^ aspects of SC-islets. However, studies integrating these aspects with detailed analyses of stimulus-secretion coupling and exocytosis machinery of functional SC-islets have been lacking.

Here, we developed an optimized protocol for the generation of functional SC-islets. We compared SC-islets and primary human adult islets comprehensively to quantify systematically their similarities and differences. During the final, extended maturation stage, the cytoarchitecture of SC-islets was profoundly reorganized, and glucose-stimulated insulin secretion matured to a level similar to that of primary adult islets. We conducted detailed functional and physiological characterization of the SC-islets, supported by targeted metabolite tracing studies together with single-cell transcriptomic profiling of differentiating endocrine cell populations. This multipronged approach was conducted both during the timecourse of SC-islet maturation in vitro and after in vivo engraftment. Our integrated analyses show that a high level of beta cell functionality is achieved in vitro even if specific metabolic and transcriptomic differences persist between SC-islet beta cells and primary beta cells.

## Results

### SC-islets present organotypic cytoarchitecture and function

**Fig. 1:**
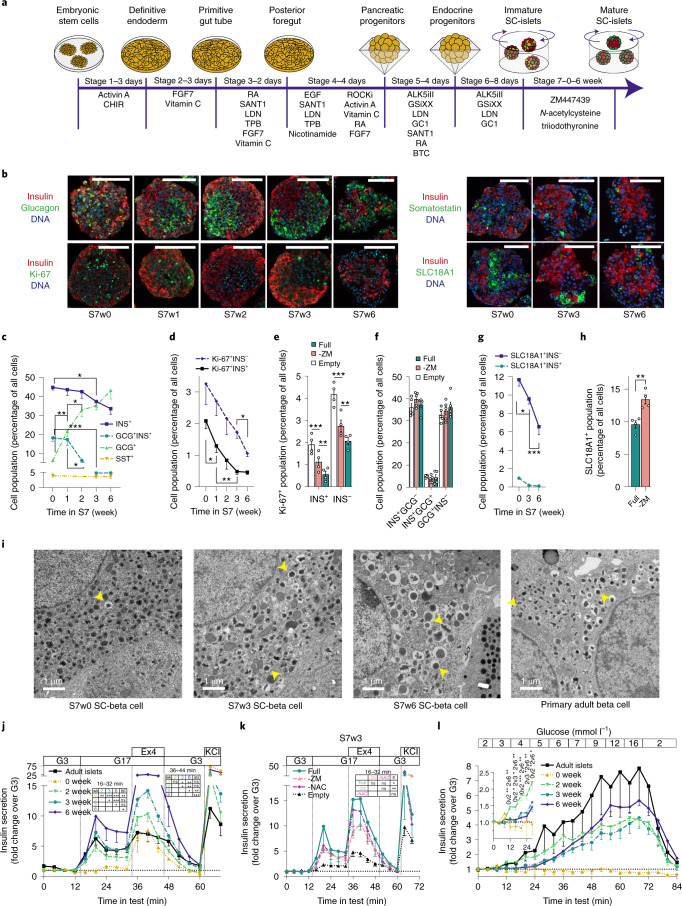
Characterization of SC-islet cytoarchitecture and insulin secretory function. **a**, Overview of SC-islet differentiation protocol. Stages 1–4 in monolayer, Stage 5 in microwells and Stages 6–7 in suspension culture. **b**, Immunohistochemistry of SC-islets during S7 culture. Scale bars, 100 µm, representative images of two to eight independent experiments with similar results. **c**–**d**, Proportion of hormone positive (**c**) and Ki-67 positive (**d**) cells during S7 culture, quantified from immunohistochemistry, *n* = 2–8. Multiple (**c**) and one-way analysis of variance (ANOVA), INS^+^ and INS^−^ populations (**d**) were analyzed separately. **e**–**f**, Percentage of S7w3 SC-islet cells positive for Ki-67 (**e**) or INS and GCG (**f**). Comparison of S7 media: ‘Full’ = ZM+NAC+T3, ‘-ZM’ = NAC+T3 and ‘Empty’ without ZM, NAC and T3; Two-way ANOVA. **g**–**h**, Proportion SLC18A1 positive cells during S7 culture (**g**) *n* = 4–5 and at S7w3 comparing full S7 medium and S7 medium lacking ZM (**h**) *n* = 4, quantified from immunohistochemistry; two-way ANOVA (**g**), two-tailed Welch’s *t*-test (**h**). **i**, Electron micrographs of SC-beta cells at S7 weeks 0, 3 and 6, and of adult human beta cells; scale bars, 1 µm. Yellow arrows denote mature insulin granules. Representative images of several cells from one to three independent experiments with similar results. **j**, Insulin secretion responses to perifusion with 2.8 mM (G3) to 16.8 mM glucose (G17), 50 ng ml^–1^ exendin-4 (Ex4) and 30 mM KCl. Normalized to secretion during the first 16 min of the test; *n* = 3–18. One-way ANOVA of the mean response during specific steps of the test. **k**, Same test as in **j**, conducted on matched S7w3 SC-islet experiments comparing Full, -ZM, -NAC (with ZM and T3) and empty S7 medium, *n* = 3–5; one-way ANOVA of the mean response during the G17 step of the test. **l**, Insulin secretion response to gradual increase in glucose concentration from 2 to 16 mM. Normalized to secretion during the first 8 min of the test. Inset: data from 0–28 min with a different *y* axis scale, *n* = 4–7, Two-way ANOVA on significance of individual timepoints of the test. All data are presented as mean ± s.e.m. *P < 0.05, **P < 0.01, ***P < 0.001.

We devised an optimized differentiation protocol by combining previous advances in the generation of SC-islets^8,9,13,14^ (Fig. 1a). Noteworthy differences to the most widespread protocols^8,9^ include: (1) differentiation of hPSCs in adherent conditions until the pancreatic progenitor stage (S4); (2) optimized S4 step including nicotinamide, epidermal growth factor, Activin A and a ROCK inhibitor^13,14^; (3) a microwell aggregation step that results in ≈ 80% PDX1^+^NKX6-1^+^ pancreatic progenitor population in uniformly sized clusters (Supplementary Fig. 1a); and (4) improved final maturation stage (S7), carried out in suspension culture. This S7 maturation step omits ALK5 inhibitor^3^ and contains an antiproliferative^15^ aurora kinase inhibitor ZM447439 (ZM) (adapted from Patent WO2017222879A1), in addition to previously described components triiodothyronine (T3) and *N*-acetyl cysteine (NAC)^9^.

To determine the impact of the S7 maturation step on SC-islet function, we systematically characterized hPSC-derived SC-islet maturation from the beginning of S7 (S7w0) to the end of its sixth week (S7w6), using a series of morphometric, functional, metabolomic and transcriptomic analyses. At S7w0, SC-islets contained ≈40% insulin-positive monohormonal cells (INS^+^)—a proportion that remained relatively stable until S7w6 (Fig. 1b,c and Supplementary Fig. 1b,c). At S7w0 and S7w1, SC-islets contained ≈15–20% cells coexpressing insulin and glucagon (INS^+^GCG^+^)—a proportion that was reduced to <5% by S7w3. Concomitantly, the number of single-positive GCG^+^ cells increased from ≈5% to ≈40–50% (Fig. 1c and Supplementary Fig. 1b), consistent with previous studies demonstrating polyhormonal to alpha cell differentiation in vitro and in vivo^7,10,11,13,16–20^. Somatostatin (SST) positive cells were present at S7w0 at around 4% and the proportion remained unchanged until S7w6 (Fig. 1c).

As functional maturation of beta cells is linked to reduced proliferation^21,22^, we examined markers of cell proliferation during the S7 and observed an 80% reduction (from 2.1% to 0.46%) in Ki-67^+^ INS^+^ cells (Fig. 1b,d). Critically, reduced proliferation was dependent on the use of ZM, NAC and T3 in S7 medium (Fig. 1e), while the proportions of INS^+^ and GCG^+^ cell populations were not affected (Fig. 1f). Stem-cell-derived enterochromaffin-like (SC-EC) cells have been reported to arise as an undesired byproduct of SC-islet differentiation^7^. We detected ≈13% SC-EC (SLC18A1^+^) cells at S7w0—a proportion that decreased steadily to ≈6.5% by S7w6 (Fig. 1g). Notably, this decrease was dependent on the presence of ZM in S7 medium (Fig. 1h).

SC-islet cytoarchitecture changed profoundly during S7 maturation. A high proportion of INS^+^ cells localized to the SC-islet periphery at S7w0–w1, but, by S7w3, SC-islets were polarized, with GCG^+^ and INS^+^ cells clustered separately (Fig. 1b). However, by S7w6, the cytoarchitecture varied from core-mantle organization (Supplementary Fig. 1d) to intermingled clusters of GCG^+^ and INS^+^ cells (Fig. 1b). Quantitatively, this reorganization resulted in an increased number of cell–cell contacts between GCG^+^ and INS^+^ cells from S7w0 to S7w6 (Supplementary Fig. 1e). Similar cytoarchitectural rearrangements have also been described during human fetal pancreatic islet development^23–25^.

While beta cell numbers remained unchanged during the first 3 weeks of S7, the insulin content of SC-islets increased fourfold (Supplementary Fig. 1f). Concurrently, SC-islet beta (SC-beta) cells progressively acquired dense core insulin granules with ultrastructural morphology resembling those of primary beta cells (Fig. 1i).

Adult primary islets are characterized by a tightly controlled, biphasic insulin secretion response to increases in glucose^26^. This is controlled through a metabolic response to glucose through K_ATP_-channel activity (the triggering pathway), and modulated through neurohormonal and metabolic amplifying pathways^27^. At S7w0, high glucose concentrations alone (16.7 mM) failed to trigger insulin secretion. However, treatment with the GLP-1 analog exendin-4 and membrane depolarization with high K^+^ both triggered pronounced secretory responses. From S7w2 onwards, SC-islets displayed biphasic glucose-stimulated insulin secretion (GSIS) responses similar to primary islets, with gradual increases in the magnitude of the response until S7w6 (Fig. 1h). Of note, the primary islets in this study had secretory responses representing the lower end of responses recorded in previous studies^28^ and in publicly available databases^29^. The SC-islets sustained their second phase response for >70 min (Supplementary Fig. 1g). The acquisition of SC-islet function was replicated in two additional human iPSC-lines (Supplementary Fig. 1h) demonstrating the robustness of the maturation protocol. Omission of either ZM or NAC from S7 medium attenuated GSIS responses, while omitting all additives nearly abolished it (Fig. 1k)—an effect mostly explained by higher insulin release in low glucose (Supplementary Fig. 1i).

Immature fetal and infantile primary beta cells are unable to suppress their insulin secretion in low glucose^30^. Beta cell functional maturity is thus reflected by the glucose concentration threshold that triggers insulin secretion^28^. Immature S7w0 SC-islets released higher levels of insulin in low glucose (Supplementary Fig. 1j). This basal release could be reduced with the K_ATP_-channel opener diazoxide (Supplementary Fig. 1k), suggesting inappropriate K_ATP_-channel closing in basal conditions at S7w0. We assessed insulin secretion thresholds also by gradually increasing glucose concentration in perifusion assays. SC-islets at S7w0 showed no glucose-induced insulin release, whereas at S7w2 they responded at unphysiologically low glucose concentrations of ≈3 mM. However, at S7w3 and S7w6 they reached the adult threshold of ≈5 mM glucose (Fig. 1l). This shift was also reflected in the glucose concentration eliciting the half-maximal secretory response, (5.6, 6.7 and 8.1 mM, at S7w2, S7w3 and S7w6, respectively) (Supplementary Fig. 1l).

These results demonstrate the generation of SC-islets in vitro, with biphasic glucose-dependent insulin secretion similar to that of adult islets. Functional maturation correlated with changes in SC-islet architecture and cell composition, but not with an increase in beta cell mass.

### Functional insulin secretion machinery in SC-beta cells

**Fig. 2:**
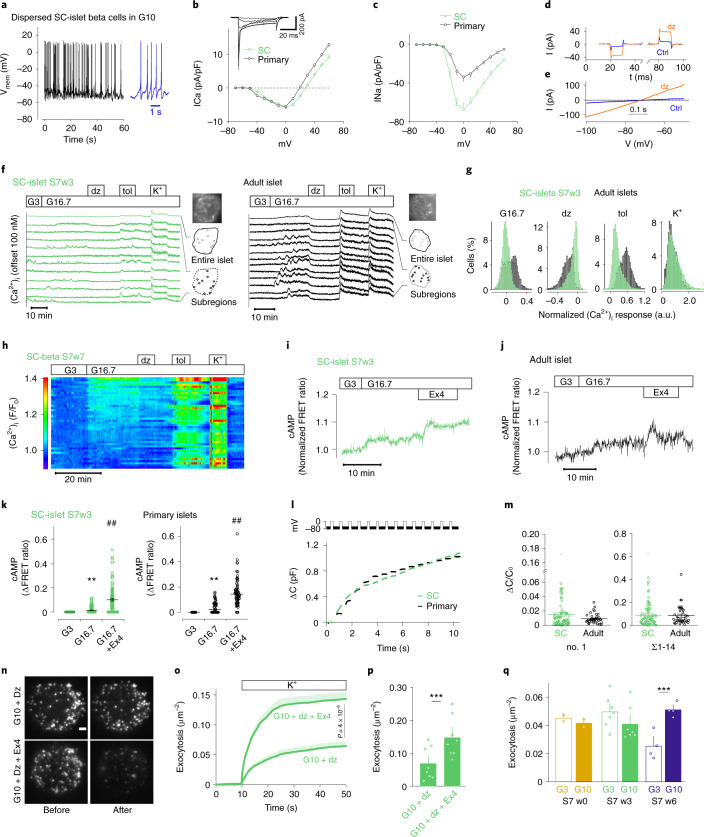
Voltage-dependent ion currents, [Ca^2+^]_i_ oscillations, [cAMP]_m_ signaling and exocytosis in SC-derived and primary islet cells. **a**, Example membrane potential recording in beta cells of dispersed SC-islets; 10 mM glucose. **b**–**c**, Current (I)–voltage (V) relationship in beta cells of dispersed SC-islets (*n* = 80 cells, eight preparations) or primary islets (*n* = 39 cells, four donors). Inset shows family of voltage-clamp currents in SC-beta cells (−40 to +10 mV). Average Ca^2+^ currents (**b**) and peak Na^+^ currents (**c**) (*P* = 0.002, two-tailed *t*-test) normalized to cell capacitance (pF). For SC-beta cells, half-maximal current activation was reached at −29 ± 0.9 mV (*n* = 64) for Ca^2+^ and at −22.5 ± 0.4 mV (*n* = 75) for Na^+^. **d**–**e**, Current responses to step-depolarizations (**d**, ±10 mV around −70 mV, black) or voltage ramps (**e**, −100 to −50 mV at 100 mV s^–1^) in controls (Ctrl) or in presence of diazoxide (200 µM) in S7w3 SC-beta cells. **f**, [Ca^2+^]_i_ recordings from SC-islets and primary islets exposed to 3 mM (G3) and 16.7 mM glucose (G16.7), 250 µM diazoxide (dz), 1 mM tolbutamide (tol) and 30 mM K^+^. The uppermost trace shows a quantification from an entire islet and the traces below are representative examples from cell-sized regions of interest. **g**, Histograms showing the changes of [Ca^2+^]_i_ in response to various treatments normalized to the levels at G3 in cells from SC-islets (*n* = 5,254) and primary islets (*n* = 3,550). **h**, [Ca^2+^]_i_ recording specifically from insulin-expressing SC-beta cells using RIP2-R-GECO1. Relative fluorescence changes as a function of time with each line representing one cell. **i**–**j**, Representative [cAMP]_m_ recordings from cells in intact SC- (**i**) and primary (**j**) islets stimulated with G16.7 and 10 nM exendin-4 (Ex4). **k**, The effects of G16.7 and Ex4 from experiments as in **i** and **j** in SC-islets (n = 119 cells from six independent experiments) and primary islets (81 cells from three preparations) ** *P* < 0.01 versus G3; ^##^ *P* < 0.01 versus G16.7, two-tailed Student’s paired *t*-test. **l**, Cell capacitance increase (ΔCm) during a train of 14 × 200 ms depolarizations from −70 mV to 0 mV in SC-beta cells and primary beta cells. **m**, Average change in membrane capacitance, normalized to initial cell capacitance (ΔC/C_0_), during the first depolarization (no. 1), and total increases during the train (Σ1–14) for SC-beta (n = 80 cells, eight preparations) and primary beta cells (*n* = 39 cells, four preparations). Dots represent individual cells and lines the mean values. **n**, Representative TIRF images of SC-beta cells expressing the granule marker NPY-tdmOrange2 in absence (top) or presence of Ex4 (bottom), and before (left) and after (right) stimulation with elevated K^+^ (in G10 + diazoxide). Scale bar, 2 µm. **o**, Cumulative timecourse of high K^+^-evoked exocytosis events normalized to cell area, from experiments as in **n**, for control (68 cells) and Ex4 (71 cells); two-tailed *t*-test. Shaded areas indicate s.e.m. **p**, Total K^+^ depolarization-induced exocytosis in **o**. **q**, Spontaneous exocytosis (normal K^+^, no diazoxide, normalized to cell area) during a 3-min observation period after >20 min preincubation at G3 or G10. Fusion events were quantified in SC-beta cells at S7w0 (13 cells at G3 and 12 at G10) and at S7w6 (40 cells at G3 and 41 at G10) and normalized to the cell area. In **p** and **q**, dots represent averages for individual SC-islet batches. All data presented as means ± s.e.m. unless otherwise indicated.

To better understand the mechanisms of SC-islet glucose sensitivity, we dissected the stimulus-secretion coupling machinery of SC-islet beta cells with measurements of ion channel conductance, cytoplasmic Ca^2+^ and cAMP concentrations, as well as exocytosis. Patch-clamp recordings showed that S7w3 SC-beta cells fired action potentials (Fig. 2a), with 11 of 16 cells active in 3 mM glucose. In S7w6 cells, 1 of 17 cells fired action potentials in 3 mM glucose, which increased to 4 active cells in 16 mM glucose. SC-beta cells had Ca^2+^- and Na^+^-currents with voltage dependences similar to those in primary human beta cells (Fig. 2b,c). Ca^2+^-current amplitude was similar in both cell types, while Na^+^-currents were about twofold larger in SC-beta cells (Fig. 2c). K_ATP_-channel dependent K^+^-conductance of S7w3 SC-beta cells was quantified using symmetric voltage-steps (Fig. 2d) or ramps (Fig. 2e). In 3 mM glucose, the membrane conductance was, on average, 53 ± 4 pS/pF (*n* = 50 cells) and increased in the presence of diazoxide in 49/50 cells to 273 ± 30 pS/pF (*n* = 50 cells). Both values are similar to those previously reported for human beta cells^31^.

The cytoplasmic Ca^2+^ concentration ([Ca^2+^]_i_) was recorded from individual cells in SC- and primary islets loaded with a fluorescent Ca^2+^ indicator. In S7w3 SC-islets, a subset of cells showed [Ca^2+^]_i_ oscillations in low glucose with little change in response to high glucose (Fig. 2f). Other cells showed low and stable [Ca^2+^]_i_ at low glucose, with increased, and often oscillatory, [Ca^2+^]_i_ in high glucose (Fig. 2f). Primary islet cells also behaved heterogeneously but a higher proportion responded to elevated glucose (Fig. 2f,g). K_ATP_-channel opening with diazoxide reduced, and closure with tolbutamide increased, [Ca^2+^]_i_ in both SC-islets and primary islets with more pronounced responses in the latter (Fig. 2f,g). Depolarization with 30 mM K^+^ increased [Ca^2+^]_i_ in all cells with similar magnitude in SC- and primary islets (Fig. 2f,g). The overall [Ca^2+^]_i_ responses or fraction of glucose-responsive cells in SC-islets did not change consistently during prolonged S7 maturation (range 42–74%; Supplementary Fig. 2a,b). However, among the glucose-responsive cells, basal [Ca^2+^]_i_ decreased from 47 ± 0.4 to 24 ± 0.2% of the K^+^-stimulated level and the increase induced by glucose stimulation improved from 5.2 ± 0.1% at S7w0 (*n* = 1,091 responsive cells) to 20.8 ± 0.4% at S7w7 (*n* = 1,659 responsive cells), consistent with the observed reduction of basal secretion and improved stimulation index. Since these unbiased analyses of indicator-loaded cells inevitably include a fraction of nonbeta cells, experiments were also performed with S7w7 SC-islets expressing the genetically encoded Ca^2+^ reporter R-GECO1 under insulin promoter control. Recordings from SC-beta cells identified by R-GECO1 expression confirmed the response heterogeneity while also highlighting that at S7w7, 79% of the beta cells showed glucose-induced [Ca^2+^]_i_ increases dependent on K_ATP_-channel closure (*n* = 130 cells; Fig. 2h and Supplementary Fig. 2c).

**Fig. 3:**
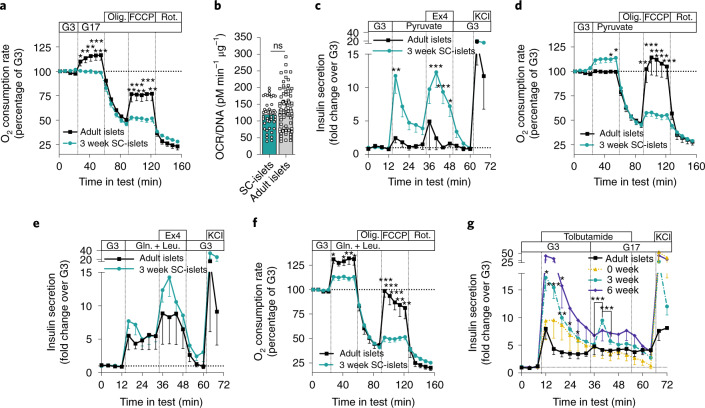
Testing of SC-islet respiration, alternative fuel responses and metabolic amplifying pathway. **a**, Change in OCR in response to 16.8 mM glucose (G17), oligomycin (Olig.) (2 µM), FCCP (2 µM) and rotenone (Rot.) (1 µM) in S7w3 SC-islets (*n* = 15) and adult islets (*n* = 5); two-tailed Student’s unpaired *t*-test. **b**, OCR normalized to DNA content of the SC-islets in **a**; two-tailed Student’s unpaired *t*-test. ns, nonsignificant. **c**, Insulin secretion responses to perifusion with 2.8 mM glucose (G3), 10 mM pyruvate, 50 ng ml^–1^ exendin-4 (Ex4) and 30 mM KCl. Normalized to average secretion during, the first 16 min of the test. *n* = 3–4; one-way ANOVA of the mean response during specific steps of the test. **d**, the same test as in **a**, with pyruvate 10 mM replacing G17; two-tailed Student’s unpaired *t*-test, *n* = 4–12. **e**, Same test as **c**, with 10 mM glutamine (Gln.) and 5 mM leucine (Leu.) replacing pyruvate (*n* = 3–4). **f**, Same test as **d**, with 10 mM glutamine (Gln.) and 5 mM leucine (Leu.) replacing pyruvate; two-tailed Student’s unpaired *t*-test (*n* = 4–11). **g**, Insulin secretion responses to change from G3 to G17 under the influence of 500 µmol l^–1^ tolbutamide (Tolb) in perifusion. Normalized to secretion during the first 12 min of the test, *n* = 4–7; two-way ANOVA. Significance versus human islets, and when indicated, between timepoints in test. All data are presented as mean ± s.e.m. * *P* < 0.05, ** *P* < 0.01, *** *P* < 0.001.

In the presence of low glucose, tolbutamide increased [Ca^2+^]_i_ in both primary and SC-islets, but again to a higher degree in primary islet cells (Supplementary Fig. 2d,e). High glucose in the continued presence of tolbutamide caused a slight [Ca^2+^]_i_ increase in SC-islets and a decrease in primary islet cells. Despite this lowered [Ca^2+^]_i_, glucose amplified secretion under these conditions (Fig. 3g). Treatment with exendin-4 did not alter [Ca^2+^]_i_ in primary islets and had only a weak tendency to increase [Ca^2+^]_i_ in SC-islets (Supplementary Fig. 2d,e).

The concentration of submembrane cAMP ([cAMP]_m_)—a modulator of insulin exocytosis—was recorded in single cells in intact SC- and primary islets. High glucose induced a small, and exendin-4 a much more pronounced, increase in [cAMP]_m_ in both preparations (Fig. 2i–k) and in SC-beta cells identified by insulin-promoter-driven expression of R-GECO1 (Supplementary Fig. 2f), indicating that cAMP signaling in SC-islets closely resembles that in primary human islets.

Single-cell exocytosis was measured as membrane capacitance changes using patch clamp. A train of depolarizations (14 × 200 ms) resulted in identical capacitance increases (ΔC) of 0.087 ± 0.012 fF/pF (*n* = 80) in S7w3 beta cells and 0.084 ± 0.013 fF/pF (*n* = 39) in primary beta cells (Fig. 2l–m). Cell size, as assessed by the initial cell capacitance (Cm0), was slightly larger for SC-cells than for primary beta cells (by 27 %, *P* = 0.0001, unpaired *t*-test) (Supplementary Fig. 2f).

Docking and exocytosis of insulin granules at the plasma membrane were studied by total internal reflection (TIRF) microscopy (Fig. 2n). Depolarization of S7w0 cells with elevated K^+^ (in the presence of diazoxide to prevent spontaneous depolarization) released 0.063 ± 0.008 granules (gr) μm^−2^ (*n* = 68 cells; Fig. 2o). Exocytosis proceeded initially with a burst (4 × 10^−3^ gr μm^−2^ s^−1^, <10 s) and later decreased (to <0.7 × 10^−3^ gr μm^−2^ s^−1^). We consistently observed more than a doubling of K^+^-stimulated exocytosis following exendin-4 treatment (0.14 ± 0.01 gr μm^−2^, *n* = 71 cells; Fig. 2o,p). All exocytosis values are similar to those reported in primary beta cells^32^.

Notably, in S7w0, spontaneous exocytosis (no diazoxide) was similar in 3 mM and 10 mM glucose (0.045 ± 0.004 gr μm^−2^, *n* = 13 versus 0.041 ± 0.002 gr μm^−2^, *n* = 12) (Fig. 2q). In contrast, at S7w6, basal exocytosis was lower (0.025 ± 0.002 gr μm^−2^, *n* = 40) and doubled when glucose was raised to 10 mM (0.051 ± 0.003 gr μm^−2^, *n* = 41; Fig. 2q).

The density of docked granules was ~ 0.6 gr μm^−2^ in S7w3 cells (Supplementary Fig. 2g–k), which is identical to values reported for primary beta cells^32^. Treatment with exendin-4 slightly increased docked granules when exocytosis was prevented with diazoxide (Supplementary Fig. 2g).

In summary, these analyses showed that SC-beta cells are equipped with the necessary ion channels, exocytosis components and intracellular signaling machinery required for fine-tuned regulation of insulin secretion.

### SC-islets exhibit immature mitochondrial glucose coupling

As SC-islets display functionally mature exocytotic machinery, we next sought to uncover the extent of metabolic coupling to insulin release. Glucose-induced mitochondrial respiration is another characteristic feature of functional adult islets, which correlates with GSIS^2,33–35^. We assayed oxygen consumption rate (OCR) during glucose stimulation (Fig. 3a) and observed that glucose increased mitochondrial respiration in primary islets but not in SC-islets, despite similar insulin secretion dynamics (Fig. 1j). This lack of respiratory response to glucose was not explained by aberrantly low or high basal respiration rates in SC-islets (Fig. 3b). In contrast, SC-islets responded with increased respiration rates and insulin secretion to high concentrations of pyruvate, while primary islets remained unresponsive (Fig. 3c,d). This is indicative of a retention of immature metabolic characteristics in SC-islets as genes responsible for pyruvate sensitivity are ‘disallowed’ in adult islets^36^. Direct stimulation of mitochondrial metabolism using glutamine and leucine triggered similar insulin release in both primary islets and SC-islets, while the increase in respiration rates was slightly higher for primary islets (Fig. 3e,f).

Since oxidative glucose metabolism is considered essential for the activation of the triggering pathway of insulin secretion, we next sought to clarify if a compensatory metabolic amplifying pathway may help explain SC-islet function despite the low oxidative metabolic response. We therefore exposed SC-islets and adult islets to high glucose under tolbutamide stimulation to determine the degree of insulin secretion occurring independently from K_ATP_-channel closure. S7w3 and w6 SC-islets demonstrated a stronger initial insulin secretion response to tolbutamide than adult islets. Subsequent glucose-dependent metabolic amplification was detected in S7w3 SC-islets, but it was transient and lower compared with the initial K_ATP_-channel dependent secretion (Fig. 3g). Conversely, adult islets displayed a sustained K_ATP_-channel independent glucose-responsive amplification more similar in magnitude to that of K_ATP_-channel dependent secretion, as has been reported in previous studies^28^.

Taken together, glucose processing seems aberrant or immature in SC-islets, resulting in undetectable mitochondrial respiratory responses. However, mitochondrial activity seems intact since respiration increases and dynamic insulin release can be elicited with other direct mitochondrial substrates, suggesting this discrepancy is not due simply to a low proportion of beta cells in SC-islets. Glucose-dependent insulin secretion independent of the K_ATP_-channel is weakly present in SC-islets, suggesting metabolic amplification is a minor factor in explaining the discrepancy between robust insulin secretion and weak glucose-responsive respiration.

### SC-islets demonstrate an immature glucose metabolism

**Fig. 4:**
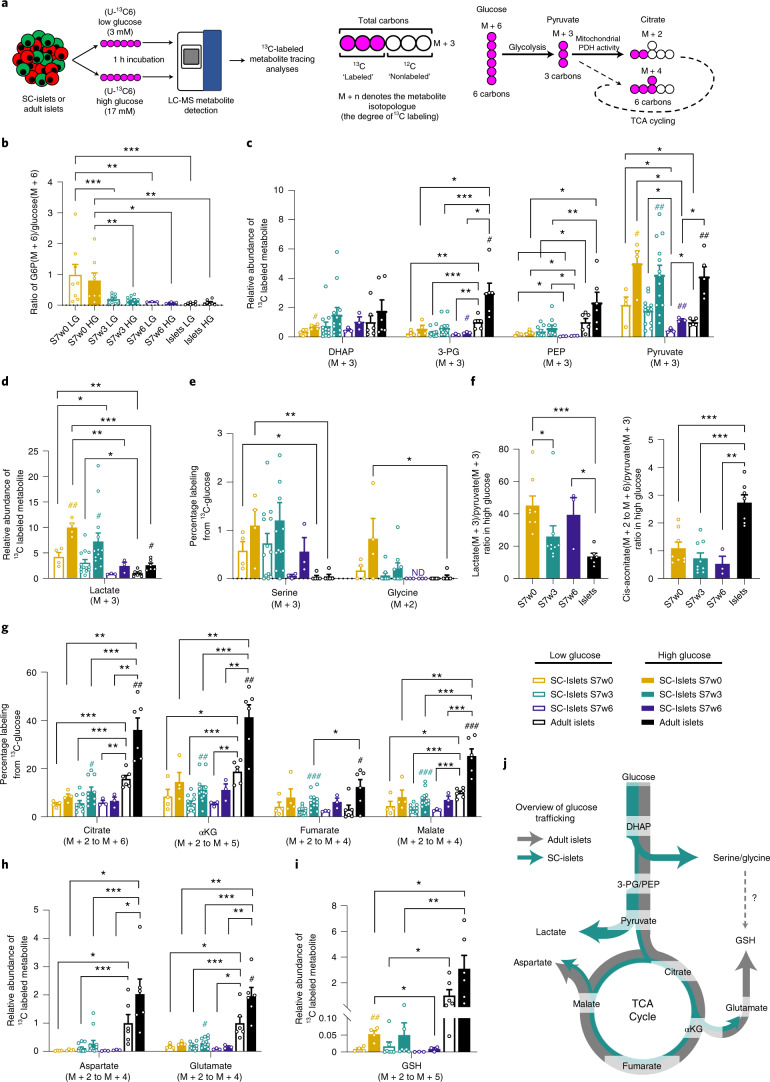
Metabolic tracing analysis of maturing SC-islets. **a**, *Left*, Overview of experimental setup. SC-islets or adult islets were exposed to low (3 mM) or high (17 mM) concentrations of uniformly labeled [U-^13^C6] glucose for 1 h before metabolite extraction and liquid-chromatography mass spectrometry (LC-MS) detection. *Right*, An example of isotopologue nomenclature and glucose-derived labeling of downstream metabolites. **b**, The ratio of M+6 G6P to M+6 labeled glucose under low and high glucose concentrations in adult islets and SC-islets over 6 weeks of maturation. **c**, The relative abundances of fully labeled glycolytic intermediates in SC-islets and adult islets following low and high labeled glucose treatment. **d**, The M+3 lactate content of adult islets and SC-islets detected over the timecourse of maturation, following low and high labeled glucose treatment. **e**, The percentage of total serine and glycine labeled from ^13^C-glucose following low and high glucose treatment of SC- and adult islets. **f**, Ratiometric analysis of labeled lactate to labeled pyruvate, and labeled citrate to labeled pyruvate under high glucose treatment in SC-islets and adult islets. **g**, The combined percentage of labeled TCA metabolites (M+2 to fully labeled M+n) from SC- and adult islets after low and high labeled glucose treatment. **h**, The combined abundance of aspartate (M+2 to M+4) and glutamate (M+2 to M+5) isotopologues in SC-islets (w0–w6) under low and high glucose concentrations, relative to adult islets. **i**, The combined relative abundance of M+2 to M+5 GSH isotopologues under low and high labeled glucose concentrations in adult islets and SC-islets. **j**, Schematic overview of active glucose metabolic pathways in SC-islets and adult islets. Arrow thickness denotes the extent of glucose-derived carbons entering the pathway. Error bars ± s.e.m. with statistical significance determined by two-tailed *t*-tests. Hash symbols indicate internal significance from low to high glucose labeling, asterisks denote significance between SC-islet timepoints or adult islet samples at each glucose concentration. ^#,*^P < 0.05, ^##,**^P < 0.01, ^###,***^P < 0.001. SC-islets S7w0 (*n* = 4), S7w3 (*n* = 12–13), S7w6 (*n* = 3), adult islets (*n* = 6).

To further probe the discrepancy between SC-islet functionality and low respiratory coupling we investigated how glucose metabolism differed between primary islets and SC-islets. We performed metabolite tracing analyses using uniformly labeled [U-^13^C6]-glucose comparing S7w0, w3 and w6 SC-islets together with primary adult islets, under low (3 mM) and high labeled glucose (17 mM) conditions (Fig. 4a).

Beta cell glucose-sensing is mediated in part by the hexokinase step of glycolysis^34^. Over the course of SC-islet maturation, we detected a reduction in the ratio of labeled glucose-6-phosphate (G6P) to labeled glucose under both low and high glucose conditions (Fig. 4b), suggesting a tighter control of glucose uptake and phosphorylation. This pattern is also evident from the relative abundances of both labeled G6P and residual labeled glucose under low glucose conditions (Supplementary Fig. 3a). Only primary islets displayed a trend for glucose-concentration-dependent increases in the G6P/glucose ratio, which may indicate a more complete degree of regulation of the hexokinase step. We also observed reduced labeled levels of 3-phosphoglycerate (3-PG) and phosphoenolpyruvate (PEP) in SC-islets compared with primary islets, despite similar levels of labeled dihydroxyacetone phosphate (DHAP) (Fig. 4c). These results are consistent with a proposed glycolytic ‘bottleneck’ due to reduced GAPDH activity^12^. A significant decrease in the production of labeled lactate was a strong characteristic of SC-islet maturation during S7 (Fig. 4d) and is in agreement with studies that link lactate overproduction to reduced GSIS^37,38^. The diversion of 3-PG into de novo serine and glycine biosynthesis, which is low in primary islets but significantly higher in less mature SC-islets, is another possible avenue of aberrant glucose metabolism (Fig. 4e).

SC-islets showed increased labeled glucose incorporation into the core tricarboxylic acid (TCA) cycle metabolites citrate, alpha-ketoglutarate (αKG), fumarate and malate upon stimulation with high glucose, but the response was clearly lower than in primary islets (Fig. 4g). Ratiometric analyses of labeled lactate:pyruvate and labeled cis-aconitate:pyruvate further demonstrated that metabolic trafficking of pyruvate is biased towards lactate production in SC-islets, and citrate/isocitrate formation in primary islets (Fig. 4f). We inferred flux through the TCA cycle by tracing the degree of ^13^C-glucose-derived carbon incorporation into each TCA metabolite. Primary islets showed enhanced oxidative TCA cycling for citrate, αKG, fumarate and malate compared with SC-islets (Supplementary Fig. 3b–e), as well as enhanced flux through the anaplerotic pyruvate carboxylase reaction, which resulted in a high proportion of M+3 malate and fumarate isotopologues (Supplementary Fig. 3b–e). A ratiometric analysis of M+2/M+3 malate isotopologues indicated an increase in the bias towards the anaplerotic pyruvate carboxylase reaction during SC-islet maturation, towards the level seen in primary adult islets (Supplementary Fig. 3f).

Aspartate and glutamate are components of the malate–aspartate redox shuttle, a key constituent of beta cell metabolism that supports glucose-stimulated insulin secretion^39^. Primary islets used a significantly higher proportion of glucose-derived carbons to generate these amino acids than SC-islets (Fig. 4h), as well as a significantly higher amount of labeled glutathione (GSH) (Fig. 4i). In contrast, the synthesis of labeled glycine (another GSH component) was barely detectable in primary islets (Fig. 4e). Of note, primary islets maintained higher total levels of reduced and oxidized forms of glutathione and the electron carriers NAD and NADP (Supplementary Fig. 3h). This is also reflected in the glucose concentration-dependent shifts in NAD^+^/NADH ratio, which were significantly more responsive in primary islets than in SC-islets (Supplementary Fig. 3g).

We next determined the extent of glucose-dependent changes in ATP/ADP ratio, an important determinant of the K_ATP_-channel-dependent triggering pathway. SC-islets displayed a low, but nonsignificant, increase in the ATP/ADP ratio following glucose stimulation, as determined by metabolomic data and enzymatic assay (Supplementary Fig. 3i-j). In contrast, primary islets displayed a significant degree of glucose coupling to ATP/ADP ratio shifts.

Other metabolic pathways have been proposed to work in concert with the canonical oxidative phosphorylation-coupled insulin secretion model. The PEP cycle is one such model that has been suggested to function through anaplerotic regeneration of PEP from mitochondrial oxaloacetate^40^. A hallmark of such cycling is the presence of M+2 labeled PEP and pyruvate, as oxidative generation of M+2 oxaloacetate would also be used in PEP regeneration. However, we were unable to detect M+2 PEP in SC-islet or primary islet samples (Supplementary Fig. 3k), and only a low percentage of M+2 pyruvate (<5%), which is in agreement with another recent study^12^.

Reductive carboxylation of αKG to isocitrate and citrate via the IDH2 enzyme to fuel cytosolic redox reactions has been proposed recently as another mechanism of modulating insulin release in beta cells^41–43^. Using ^13^C5-glutamine labeling, we observed that such reactions do occur in SC-islets, demonstrated by the high degree of M+5 cis-aconitate enrichment, an isotopologue that could only be generated by such a reductive carboxylation reaction (Supplementary Fig. 3l). By tracking the isotopologue profile of M+3 malate, we could infer the export of citrate (or isocitrate) from the mitochondria as a component of the pyruvate–citrate, pyruvate–malate and/or glycerolipid/FFA cycle (Supplementary Fig. 3l). We detected the generation of labeled pyruvate following labeled glutamine treatment, demonstrating some degree of pyruvate regeneration from TCA metabolites (Supplementary Fig. 3l).

Thus, primary human islets and SC-islets differ not only in their core TCA cycle turnover and respiration rates under glucose stimulation, but also in the production of TCA-derived metabolites and redox pathway components. Despite the differences in both glycolytic and mitochondrial glucose metabolism (Fig. 4j), SC-islets do display dynamic glucose-sensitive insulin secretion responses.

### SC-islets control the glycemia of mice in vivo

To investigate the in vivo functional potential of immature (S7w0) and more mature (S7w3) SC-islets, we implanted them under the kidney capsule of nondiabetic mice^2,9,10,44–46^ (Supplementary Fig. 4a). Circulating human C-peptide was detectable at 1 month postengraftment in all engrafted mice. However, mice engrafted with S7w3 SC-islets demonstrated twofold higher human C-peptide levels at 2 and 3 months than S7w0 engrafted mice (Supplementary Fig. 4b). Correspondingly, blood glucose levels at 3 months were lower in S7w3 SC-islet engrafted animals (Supplementary Fig. 4c) and reached the human glycemic set point (4.5 mM) by 3 months postengraftment, as reported in primary islet engraftment studies^47^. Glucose tolerance tests showed regulated insulin secretion in response to glucose injection in mice carrying both types of grafts (Supplementary Fig. 4d), but the glucose clearance was more rapid in S7w3 engrafted mice (Supplementary Fig. 4e,f). Next, we tested whether the S7w3 SC-islet grafts could sustain normoglycemia after streptozotocin (STZ)-induced loss of endogenous mouse beta cells. Glucose tolerance tests before and after STZ treatment (after 4 months of engraftment, with assays at 5 months postengraftment) showed that both control and STZ-treated animals presented robust glucose-regulated C-peptide secretion (Supplementary Fig. 4g). Despite C-peptide levels being lower in the STZ group, the glucose levels were similarly controlled in both groups (Supplementary Fig. 4h). The proportions of INS^+^ and GCG^+^ cells in the graft were not affected by the STZ treatment (Supplementary Fig. 4i,j). After removal of the engrafted kidney, the blood glucose levels increased sharply (Supplementary Fig. 4k), demonstrating that the engrafted SC-islets were actively controlling the glycemia of the diabetic mice. Extended in vitro culture in S7 conditions thus confers SC-islets a degree of maturation that results in improved functionality upon engraftment in vivo.

### SC-islets transcriptionally mature in vitro and in vivo

**Fig. 5:**
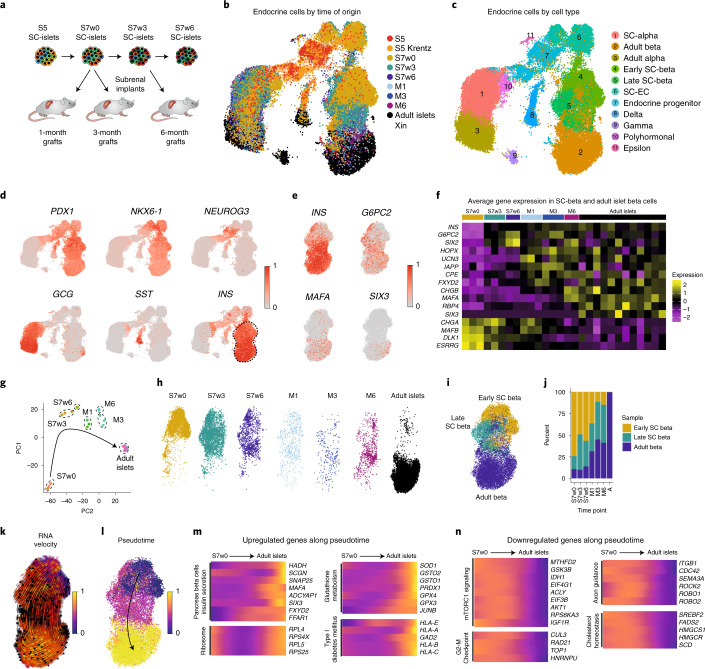
Single-cell transcriptomic profiling of stem cell derived islet cells. **a**, Experimental outline for scRNAseq transcriptomic profiling of SC-islets at the end of in vitro culture stages 5 (S5) and 6 (S7w0) and at week 3 (S7w3) and week 6 of S7 culture (S7w6), together with grafts retrieved after 1 (M1), 3 (M3) and 6 months (M6) postimplantation. **b**, UMAP-base embedding projection of an integrated dataset of 46,261 SC-derived endocrine cells and adult human islet cells^48,49^, colored by time and sample of origin. **c**, Clustering of the dataset in **b** cells into different cell types. **d**, Relative expression of marker genes for pancreatic progenitor cells (*PDX1*, *NKX6-1*, *NEUROG3*) and alpha- (*GCG*), delta- (*SST*) and beta- (*INS*) cells. Dashed line indicates the beta cell cluster selected for further study. **e**, UMAP projection of the beta cell cluster indicating the relative expression of insulin (*INS*) and mature beta cell markers *G6PC2*, *MAFA* and *SIX3*. **f**, Average gene expression of beta cell maturation markers in SC-beta cells and adult primary beta cells. The average expression of the beta cell populations (Fig. 1e) coming from each independent sample with different time of origin (S5 to Adult islets) is represented. **g**, PCA of the beta cell populations from each independent sample. (S7w0, *n* = 3; S7w3, *n* = 3; S7w6, *n* = 2; M1, *n* = 3; M3, *n* = 3; M6, *n* = 2; Adult, *n* = 12). **h**, Heterogeneous distribution of the beta cells from different time of origin in the beta cell cluster (Fig. 1e). **i**, Clustering of beta cells according to their transcriptional similarity into early, late and adult beta cluster. **j**, Fractional contribution to each early, late and adult beta clusters of beta cells from different times of origin. **k**, UMAP projection of the beta cell cluster with RNA velocity vectors overlaid. Cells are annotated by latent-time dynamics. Earlier latent timepoints, the origin of the trajectory, are indicated in blue, and later timepoints in yellow on the latent-time color scale. **l**, Pseudotemporal ordering of cells in the beta cell cluster. Earlier pseudotemporal points, the origin of the trajectory, are indicated in blue, and later pseudotemporal points in yellow on the pseudotime color scale. **m**,**n**, Relative expression levels of example genes that are upregulated (**m**) or downregulated (**n**) along the pseudotime trajectory inferred in **l**.

To investigate the transcriptional changes associated with in vitro and in vivo SC-islet maturation, we performed single-cell RNA (scRNA) sequencing on SC-islets during in vitro differentiation (S5, S7w0, S7w3 and S7w6), as well as SC-islet grafts retrieved at 1, 3 and 6 months postengraftment (Fig. 5a). We obtained a dataset comprising 38,978 cells, which we integrated with previously published datasets from S5 hPSC-derived cells (4,458 cells) and human adult islets (19,435 cells)^48,49^. The full integrated dataset had a total of 62,871 cells, including 46,261 endocrine cells that were selected for further study (Supplementary Fig. 5a–d and Supplementary Table 1; Methods).

The endocrine cell dataset was clustered according to time of origin and cell identity (Fig. 5b,c, Supplementary Fig. 5e-g and Supplementary Table 1). These populations reconstructed a differentiation continuum, from multipotent pancreatic progenitors, through intermediate differentiating stages and finally into beta (44%), alpha (33%), delta and gamma cells (Fig. 5c–d, Supplementary Tables 1 and 2 and Supplementary Fig. 5f–i). We also detected a cell population expressing FEV (a beta cell developmental transcription factor) that could represent SC-EC (Supplementary Fig. 5i). To cross-reference this and other cell types, we integrated our dataset with those described by Veres et al.^7^. Most of the cell types identified in our dataset clustered with the equivalent cell populations from the Veres et al. dataset (Supplementary Fig. 7). The cells identified as SC-EC by Veres et al. also clustered together with our FEV -expressing SC-EC cells, suggesting that they are the same cell type. However, the proportion of SC-EC cells identified in our dataset declined to undetectable levels during SC-islet maturation (Supplementary Fig. 7a).

We then focused on SC-beta cell subpopulations to determine the transcriptional changes associated with functional maturation (Fig. 5d–e and Supplementary Fig. 5j). Principal component and correlation analyses indicated that S7w3 and S7w6 SC-beta cells were transcriptionally more similar to grafted and adult beta cells than to S7w0 SC-beta cells (Fig. 5g and Supplementary Fig. 5k). We next examined the average expression of known mature beta cell marker genes across each individual sample^7,10,50–53^ (Fig. 5f). *INS*, *G6PC2* and *SIX2* gene expression increased early in in vitro culture, whereas other mature beta cell markers such as *HOPX, UCN3, IAPP, CPE* and *FXYD2* were upregulated only upon engraftment. *CHGB* and *MAFA* expression was sharply upregulated at 6 months postengraftment, suggesting the need of extended in vivo maturation for the upregulation of these genes. Interestingly *RBP4* and *SIX3* were detected primarily only in adult beta cells (Fig. 5f).

Beta cell differentiation is not a synchronous and homogeneous process, as evidenced by the coclustering of beta cells from different stages of maturation (Fig. 5h). To reduce the interference introduced by heterogeneous populations, SC-beta cells were unbiasedly clustered by transcriptional similarity, rather than by time of origin, into ‘SC-early’, ‘SC-late’ and ‘Adult beta’ categories (Fig. 5i). As expected, SC-beta cell proportions in the SC-late and Adult beta categories increased with the progression of time in vitro and in vivo (Fig. 5j). Cells in the Adult beta category presented higher expression of genes related to insulin secretion (*PCSK1, CPE, CHGB, ABCC8, FXYD2*), beta cell maturation (*MAFA, GDF15*) and oxidative phosphorylation (OXPHOS) (Supplementary Table 3).

**Fig. 6:**
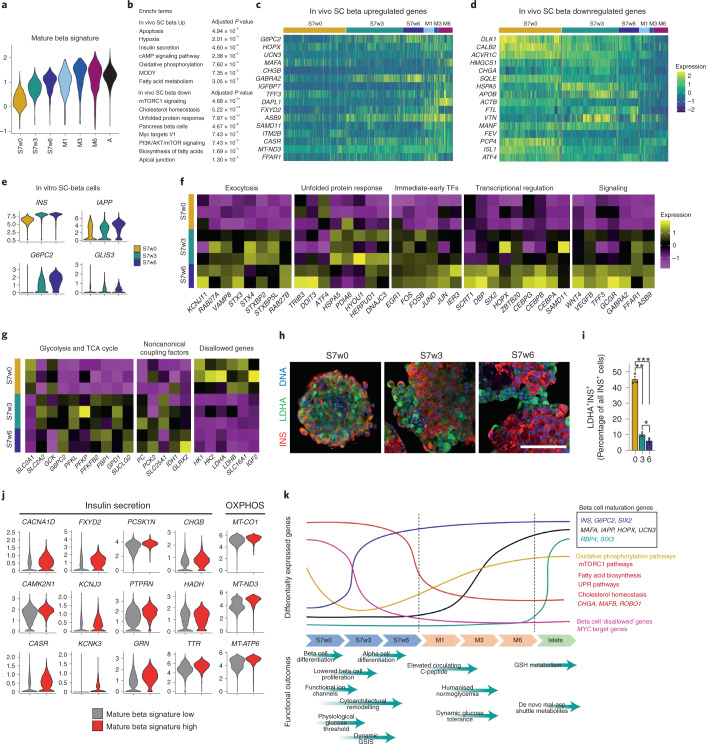
Transcriptional maturation of stem-cell-derived beta cells. **a**, Mature beta cell signature of SC-beta and adult beta cells from different times of origin. **b**, Gene sets enriched in the in vivo implanted SC-beta cells upregulated and downregulated genes compared with in vitro SC-beta cells. **c**, Expression of selected marker genes upregulated in the in vivo SC-beta cells. **d**, Expression of selected marker genes downregulated in the in vivo SC-beta cells. **e**, Violin plots representing the expression of mature beta cell markers in the SC-beta cells from S7w0, S7w3 and S7w6 times of origin. **f**, Average expression of genes associated with mature beta cell hallmark processes in individual SC-beta cell in vitro samples from different times of origin. **g**, Average expression of glucose metabolism, noncanonical coupling factors and disallowed genes in individual SC-beta cell in vitro samples from different times of origin. **h**, Immunostaining for disallowed gene LDHA protein and insulin (INS) of in vitro SC-islets from S7w0, S7w3 and S7w6 timepoints. Scale bar, 100 µm. **i**, Quantification of LDHA positive cells out of all INS positive cells in SC-islets from S7w0, S7w3 and S7w6. Data are presented as mean ± s.e.m. * *P* < 0.05, ** *P* < 0.01, *** *p* < 0.001 One-way ANOVA with Welch’s correction; *n* = 3. **j**, Expression of genes associated with insulin secretion and oxidative phosphorylation in SC-beta cells with a high or low mature beta signature. **k**, Summary of functional and transcriptomic features of SC-islet maturation in vitro and in vivo.

RNA velocity estimation (Fig. 5k) and pseudotemporal ordering (Fig. 5l) of beta cell subpopulations enabled us to infer a differentiation trajectory to investigate the genes differentially regulated upon beta cell maturation (Supplementary Fig. 5l–m and Supplementary Table 4). The expression of genes associated with pancreatic beta cell maturation and insulin secretion increased with pseudotime, together with ribosomal and HLA genes (Fig. 5m). Glutathione metabolism genes were also upregulated with pseudotime (Fig. 5m and Supplementary Fig. 5n), consistent with our metabolomic findings (Fig. 4i). Conversely, the expression of genes related to mTORC1 and MAPK signaling, cholesterol homeostasis, mitosis and MYC targets decreased with pseudotime (Fig. 5n). Genes associated with axon guidance (*ROBO1, ROBO2*) and adherens junctions were downregulated with pseudotime, indicating changes in cell migration, adhesion and cytoskeletal properties consistent with the observed cytoarchitectural changes upon maturation (Fig. 5n, Supplementary Fig. 5l–m and Supplementary Table 4). We calculated average expression levels for the genes in these processes to understand their dynamics across our dataset, showing that these mature beta program scores increased with time in vitro and in vivo, while mTORC1 and mitosis programs decreased (Fig. 6a and Supplementary Fig. 5o).

During SC-islet maturation following engraftment, SC-beta cells further upregulated genes related to beta cell maturation (*G6PC2, UCN3, MAFA*), insulin secretion (*CHGB, GABRA2*), cAMP signaling, OXPHOS and fatty acid metabolism. Whereas genes associated with mTORC1 signaling, cholesterol homeostasis and beta cell developmental transcription factors (*FEV*, *ISL1*) were downregulated (Fig. 6b–d and Supplementary Table 5). We performed an integrative analysis to understand the transcriptional differences between primary adult beta cells and the endpoints of SC-beta maturation in vitro (S7w6) and in vivo (M6) (Supplementary Fig. 5l–m and Supplementary Table 4). Glycolysis, OXPHOS and mTORC1 signaling-related genes were upregulated in more mature cells, while genes related to MAPK-, WNT- and estrogen-signaling pathways were downregulated (Supplementary Table 6 and Supplementary Fig. 6a–e). The expression of voltage-gated Na^+^ channel subunits was also downregulated upon extended maturation (Supplementary Fig. 6f), consistent with our electrophysiology findings (Fig. 2c).

### Transcriptional markers of in vitro SC-beta maturation

We then investigated the transcriptional changes specifically occurring during in vitro maturation (S7w0 to S7w6) to better understand the acquisition of in vitro function. We found that beta cell maturation markers (*IAPP*, *G6PC2*, *GLIS3*), together with exocytosis-related genes (*KCNJ11*, *RAB27A*, *VAMP8*), unfolded protein response pathway genes (*TRIB3*, *DDIT3*, *HSPA5*)^54^ and immediate-early transcription factors (*FOS, JUN*) were all significantly upregulated (Fig. 6e–f and Supplementary Table 7). Transcription factors involved in beta cell differentiation (*SIX2*, *HOPX*, *ZBTB20*)^51,55,56^ and cell cycle inhibition (*CEBP* transcription factor family^57^, and *SCRT1*^58,59^), were also upregulated in S7w6 SC-beta cells, together with ligands (*WNT4*, *TFF3*)^4,60^ and receptors associated with beta cell function (*GCGR*, *GABRA2*, *FFAR1*)^61^ (Fig. 6f). In line with the functional results, these transcriptional changes overall suggest that S7w6 beta cells present improved insulin production and exocytosis (Fig. 1j and Fig. 2q), which are associated with increased endoplasmic reticulum-stress levels and reduced proliferation (Fig. 1d)—all important hallmarks of mature beta cells^22,54^.

Glycolysis- and TCA cycle-related genes were upregulated during in vitro maturation, concomitantly with the increased expression of genes involved in noncanonical coupling processes that may act to trigger insulin exocytosis (*PC*, *PCK2*, *SLC25A1*, *IDH1*) (Fig. 6g). Contrastingly, the expression of disallowed genes such as *HK1*, monocarboxylate transporter *SLC16A1 (MCT1*) and lactate dehydrogenase isoform A (*LDHA*) were reduced during maturation in vitro (Fig. 6h–i). These results are consistent with our functional and metabolomics findings, strengthening the notion of tighter control of glycolytic flux and reduced trafficking of glucose into lactate upon SC-beta cell maturation (Fig. 1l and Fig. 4b–d).

Given the heterogeneity observed in Ca^2+^ signaling, we investigated to what extent SC-islet functionality could be driven by a subpopulation of SC-beta cells. Mature beta cell marker expression was indeed heterogeneous across SC-beta cells (Supplementary Fig. 6g). We therefore calculated a gene expression score to classify them into high and low ‘mature beta signature’ (Supplementary Table 8). Consistent with our previous analyses, genes associated with insulin secretion and OXPHOS were upregulated in the mature beta signature high cells, suggesting that this subpopulation could represent beta cells better suited for improved functionality (Fig. 6j).

We have made our single-cell datasets available via an interactive single-cell portal to facilitate the access and exploration of this resource (https://singlecell.broadinstitute.org/single_cell/study/SCP1526/).

## Discussion

Here, we describe an optimized protocol to generate human SC-islets that display glucose-sensitive insulin release and endocrine cell composition similar to that of primary islets. Moreover, through indepth functional assays, cell physiology analyses, metabolic tracing experiments and scRNA transcriptomic data throughout 6 weeks of in vitro maturation and 6 months of mouse engraftment, we show the temporal acquisition of metabolic programs and gene regulatory changes that contribute to beta cell functional maturation (Fig. 6k).

The acquisition of function observed throughout the final 6-week maturation step of in vitro differentiation correlated with a drop in markers of cell proliferation, a progressive rearrangement of SC-islet cytoarchitecture, a decrease in polyhormonal and SC-EC cell populations and a marked increase in alpha cell differentiation, all of which have been individually implicated in fetal islet development and the enhancement of mature islet function^7,10,11,13,16–20,22,62,63^.

Despite the stable number of monohormonal beta cells throughout S7 maturation, a clear physiological and transcriptomic heterogeneity is present. Only about two-thirds of SC-beta cells possess glucose-induced Ca^2+^ responses, in agreement with the scRNAseq analyses, which demonstrate that beta cell subpopulations shift in proportion throughout in vitro differentiation and in vivo engraftment. Thus, beta cell maturation does not occur synchronously at a given time, but rather consists of progressive heterogeneous changes. Targeted beta cell enrichment approaches to exploit this finding, have been used successfully to obtain more functional SC-islets^2,7^. We envision that strategies to increase the proportion of functional beta cells, on the basis of knowledge gathered from single-cell analyses such as those presented here, will pave the way to improved differentiation protocols without the need for enrichment procedures.

Perhaps the most intriguing implication of this heterogeneity is the ability of SC-islets to display islet-like functional properties in vitro, despite the large differences between adult beta cell transcriptomic profiles and metabolic coupling pathways. In contrast to primary islets, SC-islets present significantly lower mitochondrial TCA metabolite enrichment, minimal ATP/ADP and NAD^+^/NADH ratio shifts, and absent respiration spikes during glucose stimulation, all of which are key aspects of the canonical triggering pathway in functional beta cells^61^. Modulation of the K_ATP_-channel in SC-islets does show that glucose-induced Ca^2+^ influx and insulin release largely depend on K_ATP_-channel closure. Therefore, while many elements of the stimulus-secretion coupling process are functional in at least a subset of SC-beta cells, the SC-islets as a whole are not robustly coupled metabolically to the canonical triggering pathway.

To further investigate this discrepancy of in vitro SC-islet function, we used metabolite tracing to assay other proposed metabolic coupling factors leading to insulin release. Pathways, such as the PEP cycle^12,40^, the pyruvate-isocitrate cycle^41^, the pyruvate–malate cycle^64^ and the malate–aspartate redox shuttle^65^ have all been reported to contribute to the coupling of glucose metabolism to insulin secretion^39^. Our results suggest that the PEP cycle and cytosolic redox pathways may contribute, at least partly, to the robust glucose-dependent secretion response seen in SC-islets.

The tightened regulation of the hexokinase step of glycolysis, a key control point in glucose-sensing metabolism in primary beta cells, also develops over the course of in vitro SC-islet maturation, correlating with an increasing glucose concentration threshold, and lowered basal exocytosis kinetics. We also see this across metabolic and transcriptomic data, with a more stringent production of glucose-6-phosphate relative to glucose abundance, a decrease in disallowed *HK1* expression, and an increase in expression of the glucose transporter *SLC2A2* and the *G6PC2* phosphatase genes.

Despite positive functional outcomes, the maturation of beta cells is not complete in vitro. We see that, following mouse engraftment over a period of 6 months, a plethora of transcriptomic changes occur indicating continued maturation. Beta cell maturation markers such as *G6PC2* and *SIX2* are upregulated in the first few weeks of in vitro maturation. *HOPX* and *UCN3* are only upregulated briefly after implantation, whereas *MAFA* and *RBP4* display upregulation only after 3–6 months of engraftment, correlating with the humanization of mouse blood glucose levels and dynamic in vivo function. We also detect transcriptional modulation of energy-sensing mTORC1 signaling-related genes, particularly after engraftment, consistent with its reported role in postnatal mouse beta cell maturation and islet development^66–68^. This pattern is also reflected in the upregulation of mitochondrially encoded electron transport chain genes, which may indicate a stronger coupling of mitochondrial oxidative metabolism to glucose following implantation.

In summary, we present a protocol for the reliable generation of physiologically relevant SC-islets with cytoarchitecture and functionality similar to those of adult primary islets. Moreover, the multifaceted analysis that we present here constitutes a comprehensive effort to thoroughly benchmark maturing SC-islets against human primary adult islets, considered the ‘gold standard’ in the field. The combination of such integrated analyses with refined differentiation protocols will guide the generation of further improved SC-islets for the modeling of beta cell dysfunction, drug-screening purposes and cell replacement approaches, expanding both our understanding of the disease mechanisms and therapeutic possibilities to treat diabetes.

## Methods

### In vitro culture and differentiation of hPSCs

Human embryonic stem cell line H1 (WA01, WiCell) was used for most of this study. In Supplementary Fig. 1h, iPSC-lines HEL24.3 (ref. ^69^) and HEL113.5-corrected^70^ were used as well. The hPSCs were cultured on Matrigel (Corning, catalog no. 354277)-coated plates in Essential 8 (E8) medium (Thermo Fisher, catalog no. A1517001) and passaged using EDTA. To prepare the differentiation experiments, near-confluent plates of stem cells were dissociated using EDTA and seeded on new Matrigel coated plates in E8 supplemented with 5–10 μM Rho-Associated kinase inhibitor (ROCKi, catalog no. Y-27632; Selleckchem catalog no. S1049) at a density of ≈0.22 million cells cm^–2^ to achieve confluent plates. To start the differentiations, the medium was changed to D0 medium 24 h postseeding. The differentiation was carried out using a seven-stage protocol combined from key publications^8,9,13,14^ and the patent WO2017222879A1. Complete media formulations are available in the Supplementary Table 9. On the third day of S4 culture, the planar pancreatic epithelium was dissociated using 6–10 min TrypLE treatment (Thermo Fisher, catalog no. 12563029) and seeded to microwells (6-well AggreWell 400 plates, Stem Cell Technologies) at a density of 800–1,000 cells per microwell using the manufacturer’s recommended protocol. On the first day of S6 culture, the SC-islets were transferred from the microwells to suspension culture on ultralow attachment plates (Corning, catalog no. CLS3471) placed on rotator spinning at 95 r.p.m. Media changes were performed daily until the first day of S6 culture and every 2–3 days thereafter.

### In vitro culture of primary adult islets

Primary islets were provided by the Nordic Network for Islet Transplantation (Uppsala University) and University of Alberta IsletCore (Canada). They were maintained in CMRL1066 supplemented with 10% FBS, 20 mM HEPES (Gibco, catalog no. 15630-056), 2 mM Glutmax and 100 IU ml^–1^ penicillin and 100 μg ml^–1^ streptomycin on ultralow attachment plates. Islet donor characteristics are listed in Supplementary Table 10.

### Flow cytometry

Stage 4 cells and stage 7 SC-islets were dissociated with TrypLE for 5–10 min in a 37 °C water bath and resuspended in 5% FBS-containing PBS. Cells were fixed and permeabilized using Cytofix/Cytoperm (BD Biosciences, catalog no. 554714) for 20 min. Primary antibodies were incubated overnight at 4 °C and secondary antibodies for 30 min in RT in Perm/Wash buffer (BD Biosciences, catalog no. 554714) containing 5% FBS. The cells were run on FACSCalibur cytometer (BD Biosciences); data were collected with CellQuest Pro v.4.0.2 (BD Biosciences) and analyzed with FlowJo v.10 (BD Biosciences). Antibodies are listed in Supplementary Table 11.

### Immunohistochemistry and image analysis

Samples of S7 SC-islets were fixed for 2 h and samples of explanted SC-islet grafts fixed overnight in 4%PFA and embedded in paraffin. Sections (5 μm) were deparaffinized and subjected to HIER in 0.1 mmol l^–1^ citrate buffer. The slides were blocked with UV-block (Thermo Scientific, catalog no. TA-125-PBQ), and incubated with primary antibodies diluted in 0.1% Tween-20 overnight in +4 °C. Secondary antibodies were diluted similarly and incubated for 1 h at RT. Antibodies are listed in Supplementary Table 11. The slides were then imaged with Zeiss AxioImager using Apotome II with the same exposure and export setting used on all slides of each immunostaining. Images were processed in Zen2 Blue Edition v.2 (Zeiss) and analyzed using CellProfiler v.4.0 (ref. ^71^) with pipelines similar to those previously reported^70^. In brief, the nuclei were identified first and expanded along the intensity gradient of the cytoplasmic staining. Nuclei were assigned to the cytoplasm if 25% of the nuclear perimeter was overlapping with the corresponding cytoplasm. For the neighbor-to-neighbor analyses, each identified insulin+ nucleus was expanded by 35 pixels, and each overlapping insulin+ or glucagon+ or hormone negative nucleus was identified as its neighbor. The same pipeline settings were used on all images of each immunostaining, and the thresholds were set using the robust background algorithm.

### In vitro tests of insulin secretion

Static tests of insulin secretion were carried out in 1.5 ml tubes. A total of 20–30 SC-islets were handpicked and equilibrated in Krebs-Ringer buffer (KRB) with 2.8 mM glucose (G3) for 90 min, and then subjected to sequential 30-min incubations of G3, 16.8 mM glucose (G17), 0.1 µM glibenclamide (GBC) and 30 mM KCl or G3, G3 + 100 µM diazoxide, G17 + 100 µM diazoxide and 30 mM KCl in KRB. After the tests, the SC-islets were collected and the insulin and DNA contents were analyzed. Dynamic tests of insulin secretion were carried out using a perifusion apparatus (Brandel Suprafusion SF-06) with a flow rate of 0.25 ml min^–1^, and sampling every 4 min. A total of 50 handpicked SC-islets were used for each test. The SC-islets were perfused with KRB and sample collection was started after 90 min of equilibration in G3. Six different test protocols were used with conditions labeled in the corresponding figures. Insulin content of secretion fractions and SC-islet lysates was analyzed with enzyme-linked immunosorbent assay (ELISA) (Mercodia).

### Respirometry

Respiration rates of SC-islets and primary islets were measured using a Seahorse Bioscience XFe96 Extracellular Flux Analyzer and analyzed on Agilent Wave software v.2.6. S7w3 SC-islets and primary islets were loaded into Matrigel -coated Seahorse XF96 cell culture microplates (20–25 per well) and attached to the surface overnight in S7 growth medium. Before the assay, the medium was exchanged into KRB containing 3 mM glucose, and the islets allowed to equilibrate for 60–90 min at 37 °C. OCRs were measured over 150 min. Basal OCR was calculated before the sequential addition of a stimulatory nutrient (17 mM glucose, 10 mM pyruvate, or 10 mM glutamine and 5 mM leucine), an inhibitor of ATP-synthetase activity (2 µM oligomycin), a mitochondrial uncoupling agent (2 µM carbonyl cyanide-4-(trifluoromethoxy)phenylhydrazone, FCCP) and finally an inhibitor of Complex I of the electron transport chain (1 µM rotenone) in KRB. Respiration rates were normalized to the basal OCR before nutrient or small molecule addition. To compare the absolute level of OCR between SC-islets and adult islets, raw OCR values were normalized to the DNA content of each well.

### Transmission electron microscopy

Samples from SC-islets and human islets were chemically fixed with 2.5% glutaraldehyde (EM-grade, Sigma-Aldrich) in 0.1 M sodium cacodylate buffer, pH 7.4, supplemented with 2 mM calcium chloride at RT, for 2 h. After washing, the specimens were osmicated in the same buffer with 1% nonreduced osmium tetroxide on ice, for 1 h. Specimens were then washed and dehydrated in increasing concentration of ethanol and acetone, before gradual embedding into Epon (TAAB 812). After polymerization over 18 h at 60 °C, a pyramid was trimmed on the location of the embedded cells. Ultrathin, 60-nm sections were cut using an ultramicrotome (Leica ultracut UCT), picked on Pioloform-coated single-slot grids and poststained with uranyl acetate and lead citrate. Micrographs were acquired with a Hitachi HT7800 microscope (Hitachi High-Technologies) operated at 100 kV using a Rio9 CMOS-camera (AMETEK Gatan). One or two SC-islets were chosen randomly for examination and 11–12 SC-islet beta cells were selected for imaging on the basis of characteristic features of beta-like granules.

### Electrophysiology

SC-islets were dispersed into single cells in cell dissociation buffer (Thermo Fisher Scientific) supplemented with trypsin (0.005%, Life Technologies), washed and plated in serum-containing medium on 22-mm polylysine-coated coverslips, allowed to settle overnight, and then transduced with adenovirus coding for enhanced green fluorescent protein under control of the RIP2 promoter to identify beta cells.

Patch-clamp recordings were performed using an EPC-9 patch amplifier with PatchMaster v.2x90 software (HEKA Electronics). Electrodes (resistance 2–4 MΩ) were pulled from borosilicate glass capillaries, coated with Sylgard and fire-polished. Cells were superfused with an extracellular solution containing 138 mM NaCl, 5.6 mM KCl, 1.2 mM MgCl_2_, 2.6 mM CaCl_2_, 10 mM d-glucose, and 5 mM HEPES, pH 7.4 adjusted with NaOH at a rate of 0.4 ml min^–1^ at 32 °C.

Voltage-dependent currents and exocytosis were measured in whole-cell voltage-clamp mode with an intracellular solution containing 125 mM Cs-glutamate, 10 mM CsCl, 10 mM NaCl, 1 mM MgCl_2_, 0.05 mM EGTA, 3 mM Mg-ATP, 0.1 mM cAMP and 5 mM HEPES, pH 7.2 adjusted using CsOH. For current-voltage (IV) relationships, the membrane was depolarized from −70 mV to +80 mV (in 10 mV steps) lasting 50 ms each. Currents were compensated for capacitive transients and linear leak using a *P*/4 protocol. Na^+^ and Ca^2+^ current components were separated by quantifying the initial peak current (0–5 ms; Na^+^) and average sustained current (5–45 ms; Ca^2+^).

Exocytosis was quantified using the lockin module of Patchmaster (30 mV peak-to-peak; 1 kHz); with a train of 14 × 200 ms depolarizations to 0 mV at 1.4 Hz.

K_ATP_-currents were measured in whole-cell mode using a pipette solution containing 140 mM KCl, 1 mM MgCl, 10 mM EGTA, 3 mM Mg-ATP and 10 mM Hepes, pH 7.2 adjusted using KOH. The cell was held at −70 mV, and ±10 mV pulses (10 ms duration) were applied alternately at a rate of 15 Hz before and after the application of 200 μM diazoxide.

Membrane potential was measured in perforated whole-cell configuration, using a pipette solution containing 76 mM K_2_SO_4_, 10 mM KCl, 1 mM MgCl_2_ and 5 mM HEPES, pH 7.3 adjusted with KOH; access was established with amphotericin (0.25 mg ml^–1^). Glucose was varied as indicated in the text.

### Exocytosis imaging

To visualize granule exocytosis, cells treated as described for electrophysiology were additionally infected with adNPY-tdOrange2 (a well-established marker for secretory granules^72^) and imaged after 30–36 h using a custom-built lens-type TIRF microscope based on an AxioObserver Z1 with a ×100/1.45 objective (Zeiss). Excitation was from two DPSS lasers at 491 and 561 nm (Cobolt) passed through a cleanup filter (catalog no. zet405/488/561/×640x; Chroma) and controlled with an acousto-optical tunable filter (AA-Opto). Excitation and emission light were separated using a beamsplitter (catalog no. ZT405/488/561/640rpc; Chroma). The emission light was separated chromatically onto separate areas of an EMCCD camera (Roper QuantEM 512SC) using an image splitter (Optical Insights) with a cutoff at 565 nm (catalog no. 565dcxr; Chroma) and emission filters (catalog nos. ET525/50 m and 600/50 m, Chroma). Scaling was 160 nm per pixel.

Cells were imaged in a standard solution containing 138 mM NaCl, 5.6 mM KCl, 1.2 mM MgCl_2_, 2.6 mM CaCl_2_, 10 mM d-glucose, 5 mM HEPES (pH 7.4 with NaOH). Where indicated, the GLP-1 receptor agonist exendin-4 (10 nM, Anaspec) or the K_ATP_-channel opener diazoxide (200 μM, Sigma-Aldrich; to prevent spontaneous depolarizations) was also present. Where stated, exocytosis was evoked by rapidly depolarizing cells with elevated K^+^ (75 mM KCl equimolarly replacing NaCl in the standard solution, by computer-controlled local pressure ejection). Spontaneous glucose-dependent exocytosis was recorded for 3 min per cell after equilibration >20 min in the stated conditions (no diazoxide).

### [Ca^2+^]_i_ imaging

SC- and primary islets were loaded with the fluorescent indicator Fura-2 LR (ion Biosciences) by 1 h incubation with 1 µM of its acetoxymethyl ester at 37 °C in experimental buffer containing 138 mM NaCl, 4.8 mM KCl, 1.2 mM MgCl_2_, 2.56 mM CaCl_2_, 3 mM d-glucose, 25 mM HEPES (pH set to 7.40 with NaOH) and 0.5 mg ml^–1^ BSA. After rinsing in indicator-free buffer, the islets were attached to poly-l-lysine-coated coverslips in a 50-µl chamber on the stage of an Eclipse TE2000U microscope (Nikon) and superfused with buffer at a rate of 160 µl min^–1^. The chamber holder and ×40, 1.3-NA objective were maintained at 37 °C by custom-built thermostats. An LED light engine (LedHUB, Omicron Laserage Laserprodukte) equipped with 340 and 385 nm diodes and 340/26 nm (center wavelength/half-bandwidth) and 386/23 nm interference filters (Semrock, IDEX Health & Science, LLC) provided excitation light that was led to the microscope via a liquid light guide. Emission was measured at 510/40 nm using a 400 nm dichroic beamsplitter and an Evolve 512 EMCCD camera (Photometrics). Image pairs at 340/386 nm were acquired every 2 s with the MetaFluor v.7.7 software (Molecular Devices). [Ca^2+^]_i_ was calculated from the background-corrected Fura-2 LR 340/380 nm fluorescence excitation ratio from manually defined cell-sized regions of interest. The data are presented as example traces or heatmaps from individual islets and cells and as histograms of the [Ca^2+^]_i_ changes under different conditions. For unknown reasons, the apparent [Ca^2+^]_i_ values determined after in vitro calibration with the salt form of Fura-2 LR in Ca^2+^-deficient and -saturated buffers^73^ were systematically lower in SC than adult islet cells. This discrepancy could not be explained by a real difference in [Ca^2+^]_i_. To enable comparison between the preparations, the response to each treatment was calculated as the difference in time-averaged [Ca^2+^]_i_ from the preceding condition normalized to [Ca^2+^]_i_ at 3 mM glucose. The histograms show the percentage of cells at different normalized [Ca^2+^]_i_ responses using bin widths of 0.018, 0.018, 0.03 and 0.06 for glucose, diazoxide, tolbutamide and KCl, respectively, in Fig. 2g, and 0.015, 0.01, 0.012 and 0.03 for tolbutamide, tolbutamide with high glucose, exendin-4 and KCl in Supplementary Fig. 2f. For comparison of the basal [Ca^2+^]_i_, the signal was normalized against that at high KCl. All calculations were made using built-in functions of the Igor Pro 8 software (Wavemetrics).

We performed a series of experiments with SC-islets transduced with adenovirus vector expressing the Ca^2+^ reporter protein R-GECO1 under control of the rat insulin promoter RIP2. The islets were allowed to express the protein for 48 h before microscopy analysis. Infected islets were preincubated for 1 h in experimental buffer. We performed imaging as described above but using a 505–600-nm LED (Omicron Laserage Laserprodukte) and a 561/2 nm filter for excitation (Semrock), a zt405/488/561/640rpc-UF2 dichroic mirror (Chroma Technology Group) and a 609/62 nm filter (Semrock) for emission. The R-GECO1 data is presented as the fluorescence intensity, F, normalized to the initial fluorescence, F0. The heatmaps in Fig. 2 and Supplementary Fig. 2 show F/F0 (R-GECO1) and [Ca^2+^]_i_ (for Fura-2 LR) as a function of time, with each line representing a single cell. Glucose-responsive cells were defined as cells in which the difference in time-averaged [Ca^2+^]_i_ signals between high and low glucose conditions >0.

### [cAMP]_m_ imaging

SC- and primary islets were transduced with adenovirus expressing the FRET-based cAMP reporter Epac-S^H188^ (ref. ^74^) and cultured overnight. Immediately before imaging, the islets were incubated for 30 min in similar experimental buffer as for the [Ca^2+^]_i_ recordings. The islets were subsequently placed onto a polylysine-coated coverslip that was used as an exchangeable bottom of an open 50 µl chamber superfused with buffer at 200 µl min^–1^. The chamber was mounted on the thermostated stage of a TIRF imaging setup based on an Eclipse Ti (Nikon) microscope with a ×60, 1.45-NA objective. A 445-dnm diode laser (Cobolt AB) was used for excitation of the FRET donor and emission was measured at 483/32 and 542/27 nm (Semrock) with an EMCCD camera (DU-897, Andor Technology). The filters were mounted in a filter wheel (Sutter Instruments), which, together with the camera was controlled by MetaFluor software. [cAMP]_m_ is expressed as the background-corrected 483/542 nm emission ratio (FRET ratio) extracted from cell-sized regions of interest. The experiments for Supplementary Fig. 2g were performed with cells coinfected with RIP2-R-GECO1 allowing identification of SC-beta cells.

### Metabolite tracing analysis

For metabolite tracing assays, 200 SC-islets or primary islets were used for each technical replicate of ^13^C_6_-glucose labeling or ^13^C_5_-glutamine labeling. Islets were counted into wells of a 12-well tissue culture plate in a volume of 1 ml KRB containing 3 mM unlabeled glucose. Islets were then incubated on a rotator plate at 95 RPM for 90 min at 37 °C and 5% CO_2_ before being transferred to Eppendorf tubes and the basal KRB exchanged for a 0.9 ml volume of KRB containing either 3 mM (low) or 17 mM (high) [U-^13^C_6_] glucose (Cambridge Isotope Laboratories, catalog no. CLM 1396). For glutamine-labeling experiments, islets were instead incubated in either 2 mM (low) or 10 mM (high) [U-^13^C_5_] glutamine (Cambridge Isotope Laboratories, catalog no. CLM 1822). The high glutamine condition was also supplemented with 5 mM unlabeled leucine (Sigma). Islets were then incubated for 1 h at 37 °C and 5% CO_2_. After incubation, islets were washed in cold PBS before cell lysis and metabolite extraction in 75 μl of lysis buffer (80% acetonitrile in dH_2_O). Islets were lysed with mild trituration before centrifugation at 10,000g for 10 min at 4 °C. Supernatant was transferred into Chromacol (03-FISV) MS vials with a 300 µl glass insert (Thermo Fisher) and sealed with Chromacol caps with white presplit septa (Thermo Fisher), and the remaining cell pellet used for DNA quantification. Samples were analyzed on a Thermo Q Exactive Focus Quadrupole Orbitrap mass spectrometer coupled with a Thermo Dionex UltiMate 3000 HPLC system (Thermo Fisher Scientific). The HPLC was equipped with a hydrophilic ZIC-pHILIC column (150 × 2.1 mm, 5 μm) with a ZIC-pHILIC guard column (20 × 2.1 mm, 5 μm, Merck Sequant). A 5 μl aliquot of each sample was used for each assay. Metabolite separation was achieved by applying a linear gradient of organic solvent (80–35% acetonitrile, 20 mM ammonium bicarbonate) at 0.15 ml min^–1^ for 16 min at 45 °C. Metabolites were analyzed using heated electrospray ionization (H-ESI) with polarity switching (3,400 V for positive, 3,000 V for negative) at 280 °C, with ion transfer at 300 °C. Xcalibur v.4.1.31.9 software (Thermo Scientific) was used for LC-MS control. Confirmation of metabolite peak specificity was achieved using commercially available standards (Merck, Cambridge Isotope Laboratories and Santa Cruz Biotechnology). LC-MS data quality was monitored throughout the run with running standard mixes, inhouse quality controls and blanks for detecting carry over. Peak integration and metabolite isotopologue identification was accomplished using TraceFinder SP2 software v.4.1 (Thermo Scientific). Specificity of labeled peaks and isotopologues were confirmed using cell line controls, blank control samples and nonlabeled islet samples pre- and postincubation. Natural abundance was assayed using nonlabeled cell samples, and confirmed with correction calculations using IsoCor software on a subset of data^75^. To avoid any possible confounding effect of naturally occurring M+1 labeling, M+1 isotopologues were omitted from the final analyses of the relevant metabolites. Each metabolite peak area was normalized to the cell lysate DNA content or calculated as a percentage of the total M+0 (nonlabeled) and M+>1 (labeled) metabolite present in the sample. DNA normalization was also in strong agreement with levels of essential amino acids (that could not be metabolized from glucose) in each sample (data not shown). Relative abundance values are presented relative to the normalized metabolite level in primary adult islet samples in low (3 mM) [U-^13^C_6_] glucose. For NAD^+^/NADH and ATP/ADP ratio data, values were calculated from total normalized abundances in low and high glucose conditions.

### ATP/ADP ratio assay

Glucose-responsive ATP/ADP ratios were measured using the Bioluminescent ATP/ADP Ratio Assay Kit (Merck MAK135) following a modified version of the manufacturer’s protocol. Briefly, from three to five SC-islets were added to 40 µl of either low (3 mM) or high (17 mM) glucose in KRB in each well of a 96-well plate (white-walled, clear bottom) (Thermo Fisher Scientific). After 15 min of incubation at 37 °C, 90 µl of ATP reaction mix was added per well and incubated for 1 min (Measurement A). Luminescent measurements were taken using a EnSpire Plate Reader (2,300/455). After a 20-min incubation at room temperature, a second measurement was taken (Measurement B), before the addition of 5 µl of ADP reaction mix per well. A third and final measurement was taken after another 1-min incubation (Measurement C). ATP/ADP ratio was calculated as Measurement A/(Measurement C – Measurement B).

### Animal experiments

Animal care and experiments were approved by National Animal Experiment Board in Finland (ESAVI/14852/2018). NOD-SCID-Gamma (NSG, Jackson Laboratories, catalog no. 0055577) mice were housed in the Biomedicum Helsinki conventional facility in 12 h light/dark cycle and fed standard chow. For SC-islet implantations, 250–750 SC-islets (diameter 100–200 μm), were aspirated into PE-50 tubing and compacted by centrifuging. Mice were anesthetized with isoflurane and the kidney exposed. A small opening to the kidney capsule was made and the capsule separated with a glass rod. The tubing was inserted in the opening and the SC-islets implanted using a Hamilton syringe. The kidney capsule was then closed by cautery before wound closure. Subcutaneous carprofen 5 mg kg^–1^ (Zoetis) and buprenorphine 0.05–0.1 mg kg^–1^ (RB Pharmaceuticals) were used as analgesics. Nonfasted blood samples were collected from the saphenous vein monthly. Some mice received a single high-dose streptozotocin (STZ, catalog no. S0130, Sigma-Aldrich) injection (130 mg kg^–1^) 4 months after engraftment to eliminate the mouse beta cells. To verify the functionality of the SC-islet graft, the engrafted kidney was removed 1 month after the STZ injection after ligating the renal vein and artery and the ureter and removing the entire kidney. To test the functionality of the SC-islet grafts, the mice were subjected to an intraperitoneal glucose tolerance test. Mice were fasted 6–8 h before the test. The mice were weighed, and blood glucose was measured before the test. Glucose (3 g kg^–1^) was injected intraperitoneally, and blood samples (30 μl) were taken from the saphenous vein after 15 min, 30 min, 60 min and 90 min to measure blood glucose and circulating human C-peptide levels by ELISA (Mercodia).

### scRNA sequencing sample preparation

SC-islets were incubated with 2 ml of a 1:1 mixture of TrypLE Select (Thermo catalog no. 12563-029) and Trypsin-EDTA (Sigma catalog no. T4174, 10× stock diluted 1:10 with PBS) for 10 min at 37 °C in a 15 ml Falcon tube. The tube was swirled constantly and the suspension pipetted up and down after 5 min and at the end of the incubation until a single-cell suspension was achieved. Dissociation medium was neutralized by adding 12 ml of ice-cold 5% FBS-PBS, cells were mixed by inverting the falcon tube up and down. Dissociated cells were passed through a 30 μm strainer (BD) to remove cell clumps, centrifuged at 200 r.c.f. for 5 min, washed twice in encapsulation buffer (1× PBS with 0.04% BSA), counted and adjusted to a 1 × 10^6^ cells ml^–1^ cell suspension for encapsulation. To prepare single-cell suspensions from grafted SC-islet cells, grafts were first carefully retrieved from dissected kidneys. Kidney capsule was gently removed and graft tissue was scraped with a scalpel, avoiding the kidney parenchyme. Recovered graft tissue was then minced with a scalpel in small pieces and dissociated into single cells as described above. Single cells from in vitro and graft-recovered SC-islets were encapsulated using the 10x Genomics Chromium platform.

### scRNA sequencing analysis

Single-cell gene expression profiles were generated with 10x Genomics Chromium Single-Cell 3′ RNAseq platform using the Chromium Next GEM Single-Cell 3′ Gene Expression (v.3.1 chemistry). This resulted in 220 M read pairs on average per sample (with 26–28 bp read 1, 8 bp i7 index and 89–98 bp read 2). We included two reference scRNAseq datasets in our analysis; 1 sample of pancreatic endocrine progenitor cells^48^ and 12 samples of primary adult human pancreatic islets^49^. We performed raw data (fastq) processing with 10x Genomics Cell Ranger (v.3.1) pipeline. The reads were mapped to a hybrid of human and mouse reference genomes (GRCh38.98 and GRCm38.98). The unique molecular identifier (UMI) counts were filtered with DropletUtils^76^ to remove empty droplets (false discovery rate ≥0.01), mouse cells and mouse transcripts from the data. The filtered counts were analyzed with Seurat^77^. The counts were normalized, scaled and analyzed for principal component analysis (PCA) with default methods. The variable genes (top 1,000) were identified separately for each sample and combined during the analysis (for a total of 6,900 variable genes). To reduce biases among datasets we used Harmony^78^ on the first 50 PCs with sample as the covariable (with theta = 2, nclust = 50, max.iter.cluster = 40, max.iter.harmony = 10). The integrated (harmonized) principal components (PC) were used to build the uniform manifold approximation and projection (UMAP), find the neighboring cells (using shared nearest neighbor) and identify cell clusters using default Seurat methods. To reduce background RNA contamination from disrupted cells we used SoupX^79^ with clusters identified with Seurat, and known cell type specific marker genes (*GCG*, *TTR*, *INS*, *IAPP*, *SST*, *GHRL*, *PPY*, *COL3A1*, *CPA1*, *CLPS*, *REG1A*, *CELA3A*, *CTRB1*, *CTRB2*, *PRSS2*, *CPA2*, *KRT19*, *VTCN1*) to estimate the level of contamination. The Seurat analysis was then repeated with the adjusted counts with the following modifications. Cells with less than 1,000 UMI counts or 200 expressed genes were excluded. We also removed cells with unusually high level of mitochondrial reads (>25% of counts). During clustering the resolution was adjusted to 0.2. The clusters were reordered by similarity and identified to cell types by the differentially expressed genes corresponding to known marker genes. We focused the analysis on the identified endocrine cells by selecting clusters that expressed endocrine markers CHGA, INS, GCG or SST. We then rebuilt the UMAP and clustering on those cells. To improve gene expression representation we used a denoising and imputation method with Rmagic^80^. Differentially expressed genes among clusters and sample types were identified with the *FindMarkers* function in Seurat, using MAST^81^, with a log fold-change threshold of 0.15 and statistical cutoff of adjusted *P* value <0.05. We inferred the differentiation trajectories of the beta populations using RNA velocity. The reads mapping to exons and introns were recounted with velocyto^82^ and analyzed with scVelo^83^, which was used to infer RNA velocities using ‘dynamical’ mode, recover ‘latent-time’ dynamics and embed them into UMAP projection. We performed pseudotime analysis on the beta cell populations cells using Monocle2 (ref. ^84^). The data was reanalyzed using *clusterCells* to identify the different timepoint populations. The 2,000 genes with lowest q-value identified with *differentialGeneTes*t function were used for dimensionality reduction using DDRTree in the *reduceDimension* function. Pseudotime heatmaps were generated with the *plot_pseudotime_heatmap* function for the top 1,500 genes differentially expressed along pseudotime trajectory identified using *differentialGeneTest* function. The list of genes can be found in Supplementary Table 4. Aggregated signature gene scores were calculated using *AddModuleScore* function in Seurat. MSigDB_Hallmark_2020 gene lists were used to calculate scores for OXPHOS, mTORC1, MYC targets and G2–M checkpoint signatures. The following mature beta cell marker genes were used to calculate the mature beta signature: *INS*, *G6PC2*, *HOPX*, *UCN3*, *IAPP*, *CHGB*, *MAFA* and *SIX3*. We performed gene set enrichment analysis using Enrichr^85^ with a adjusted *P* value cutoff of <0.05, PANTHER v.16 and Metascape v.3.5 using the Kyoto Encyclopedia of Genes and Genomes Pathway and Hallmark Gene Sets^86^.

### Comparison with Veres et al. 2019 dataset

To compare the SC-derived cell populations described by Veres et al.^7^ with our dataset, we had to skip the initial analysis steps involving with read mapping, droplet identification and RNA contamination removal. Although we would ideally have liked to use the same preprocessing steps as in our analysis, the dataset of Veres et al. was produced with a different scRNAseq method (inDrops), which presents variable barcode location and higher error rate^87^, precluding the use of Cell Ranger for mapping and read counting. Also, the extremely high level of ambient RNA contamination (>70%) precludes using SoupX^79^. Instead we used the read counts (UMI) and metadata provided in the GEO submission (GSE114412) as a starting point for the comparison. Since the analysis by Veres et al. used a different genome annotation, we had to exclude genes that were not included in both datasets (retaining 19,170 shared genes). We combined the datasets with those of Seurat^77^ and normalized the expression with default settings. The variable genes (top 1,000 per sample) were identified separately for each sample, the data was scaled, and the top 50 PCs were identified with default settings. The resulting datasets were harmonized with Harmony^78^ using sample ID as grouping variable, theta set to 2, using 50 clusters and maximum iterations per cluster set to 40 and maximum iterations for harmony set to 10. The harmonized PCA values were used as input for UMAP, and the UMAP values were used to identify neighboring cells with default settings. Clustering was carried out at resolution set to 2. Clusters that were highly correlated with previously identified cell types were identified using clustifyr^88^. Some clusters were annotated manually as we had excluded the nonendocrine cells for our endocrine cell dataset.

### Data collection and statistical methods

Morphological data represents population-wide observations from independent differentiation experiments. Insulin secretion, respirometry and metabolomics data represents samples of independent SC-islet differentiation experiments or islet donors. Electrophysiology and measurements of Ca^2+^, cAMP and exocytosis represent recordings from individual cells in independent experiments, which are pooled depending on the differentiation experiment, or represented as individual measurements. In vivo data is derived from independent animals. Transcriptomics data represents data on the level of single cells, which are pooled from two to three independent differentiation experiments per timepoint. Statistical methods used are represented in each figure legend.

### Reporting Summary

Further information on research design is available in the Nature Research Reporting Summary linked to this article.

## Online content

Any methods, additional references, Nature Research reporting summaries, source data, extended data, supplementary information, acknowledgements, peer review information; details of author contributions and competing interests; and statements of data and code availability are available at 10.1038/s41587-022-01219-z.

## Supplementary information

Supplementary Information Supplementary Figs. 1–7 and Tables 9–11.

Reporting Summary.

Supplementary Table 1 Cell identity quantification of single cells passing quality control in the integrated full and endocrine filtered dataset.

Supplementary Table 2 Marker genes of each cluster and cell type in the endocrine cell filtered dataset.

Supplementary Table 3 Marker genes of ‘SC-early’, ‘SC-late’ and ‘Adult’ beta cell identity categories and gene set enrichments.

Supplementary Table 4 List of genes differentially expressed across beta cell maturation pseudotime.

Supplementary Table 5 Differentially expressed genes between in vitro and in vivo SC-islet beta cells.

Supplementary Table 6 Differentially expressed genes between endpoint in vitro maturation, endpoint in vivo maturation and adult beta cells.

Supplementary Table 7 Marker genes of SC-islet beta cells from S7w0, S7w3 and S7w6 timepoints.

Supplementary Table 8 Differentially expressed genes between in vitro SC-beta cells with Mature High signature versus Mature Low signature.

## Data Availability

All scRNA sequencing data are deposited in the Gene Expression Omnibus database under accession code GSE167880, and additionally on an interactive single-cell portal (https://singlecell.broadinstitute.org/single_cell/study/SCP1526). All other data are available upon reasonable request from the corresponding author.
