# Supplementary material for: Functional, metabolic and transcriptional maturation of human pancreatic islets derived from stem cells
Source: Nat Biotechnol. 2022 Mar 3;40(7):1042–55. doi: 10.1038/s41587-022-01219-z (PMC9287162; doi:10.1038/s41587-022-01219-z)
Supplement: Supplementary file 1 — Supplementary Figs. 1–7 and Tables 9–11. [file 41587_2022_1219_MOESM1_ESM.pdf]

nature portfolio

https://doi.org/10.1038/s41587-022-01219-z

Supplementary information

Functional, metabolic and transcriptional maturation of human pancreatic islets derived from stem cells

In the format provided by the authors and unedited

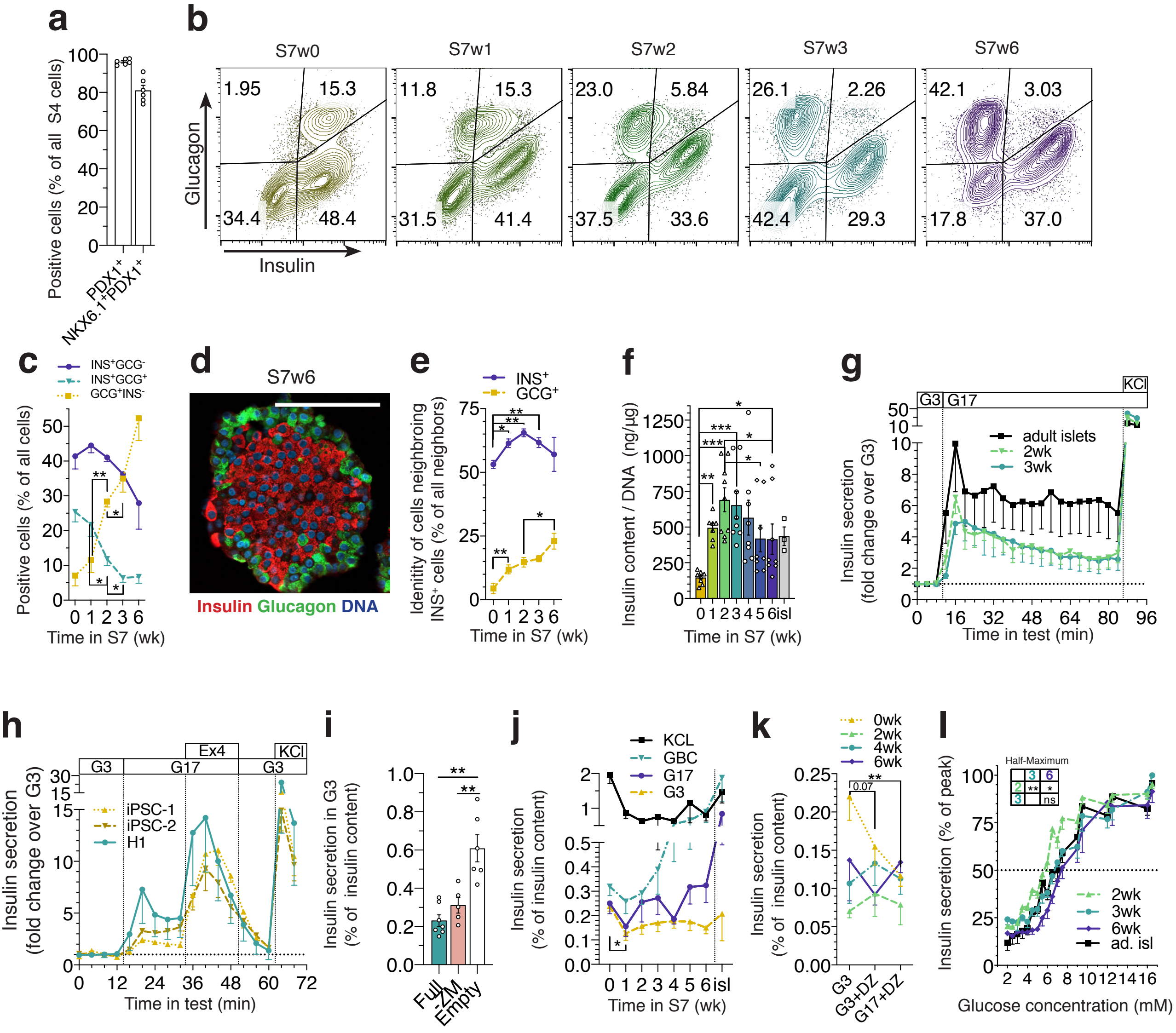

a
b
S7w0
S7w1
S7w2
S7w3
S7w6
Glucagon
Insulin
Positive cells (% of all S4 cells)
PDX1+
NKX6.1+PDX1+
c
INS+GCG-
INS+GCG+
GCG+INS-
Positive cells (% of all cells)
Time in S7 (wk)
d
S7w6
Insulin Glucagon DNA
e
INS+
GCG+
Idenitity of cells neighboring INS+ cells (% of all neighbors)
Time in S7 (wk)
f
Insulin content / DNA (ng/μg)
Time in S7 (wk)
g
G3
G17
KCl
adult islets
2wk
3wk
Insulin secretion (fold change over G3)
Time in test (min)
h
G3
G17
Ex4
G3
KCl
iPSC-1
iPSC-2
H1
Insulin secretion (fold change over G3)
Time in test (min)
i
Insulin secretion in G3 (% of insulin content)
Full
-ZM
Empty
j
KCL
GBC
G17
G3
Insulin secretion (% of insulin content)
Time in S7 (wk)
k
0wk
2wk
4wk
6wk
Insulin secretion (% of insulin content)
G3
G3+DZ
G17+DZ
l
Half-Maximum
2wk
3wk
6wk
ad. isl
Insulin secretion (% of peak)
Glucose concentration (mM)

Supplementary Figure 1

(a) Quantification of flow cytometry for PDX1 and NKX6.1, conducted at S4 after the adherent-to-microwell dissociation.

(b) Representative flow cytometry for INS and GCG at stage 7

(c) Quantification of the populations in (a), n=3-7, Two-way ANOVA.

(**d**) Immunohistochemistry representing a core-mantle organized S7w6 SC-islet, the other extreme in the spectrum of cytoarchitecture at S7w6, n=2, scale bar = 100 µm.

(e) INS^+ and GCG^+ nuclei within 8 µm of INS^+ nuclei, percentage of all nuclei within 8 µm of INS^+ nuclei. Quantified from INS – GCG immunostainings. n=2-9, population-wide characterizations of individual differentiations. Two-way ANOVA.

(f) Insulin content normalized to DNA content of the lysed SC-islets after static incubations (Supp.Fig.1j), One-way ANOVA with Welch's correction

(g) Insulin secretion response to 72-minute stimulation with 17mM glucose (G17). Normalized to secretion during 3mM glucose (G3) in the first 12 minutes of the test. n=3-4.

(h) Insulin secretion responses of S7w3 SC-islets to stimulation from 2.8 mM to 16.8 mM glucose (G3 to G17), 50 ng/ml exendin-4 (Ex4) and 30 mM KCl in perifusion. Comparison of two wildtype iPSC lines (n=2-3) and H1 hESC-line used in the rest of the study (n=18)

(i) Insulin secretion in G3 as percentage of total insulin content during a 30-minute incubation in a static insulin secretion assay, comparison of different media compositions used throughout S7. One-way ANOVA with Welch's correction.

(**j**) Insulin secretion as percentage of total insulin content during 30-min incubations in G3, G17, 0.1 µM K_{ATP}-channel closing glibenclamide (GBC) and 30 mM KCl in a static test, n=3-8 for SC-islets, n=3 for primary islets, t-test with Welch's correction.

(k) Insulin secretion as percentage of total insulin content during 30-min incubations in G3, G3 + K_{ATP}-channel opener diazoxide 100 µM (DZ) and G17 + DZ in a static test, n=3-7, Two-way ANOVA.

(**l**) Data in Figure 1l alternatively quantified as percentage of maximal secretion, plotted against the glucose concentration, statistics matrix shows significance for comparisons of the glucose concentration eliciting half-maximal secretion, n=4-6, One-way ANOVA.

All data are presented as mean ± SEM. * $p < 0.05$, ** $p < 0.01$, *** $p < 0.001$

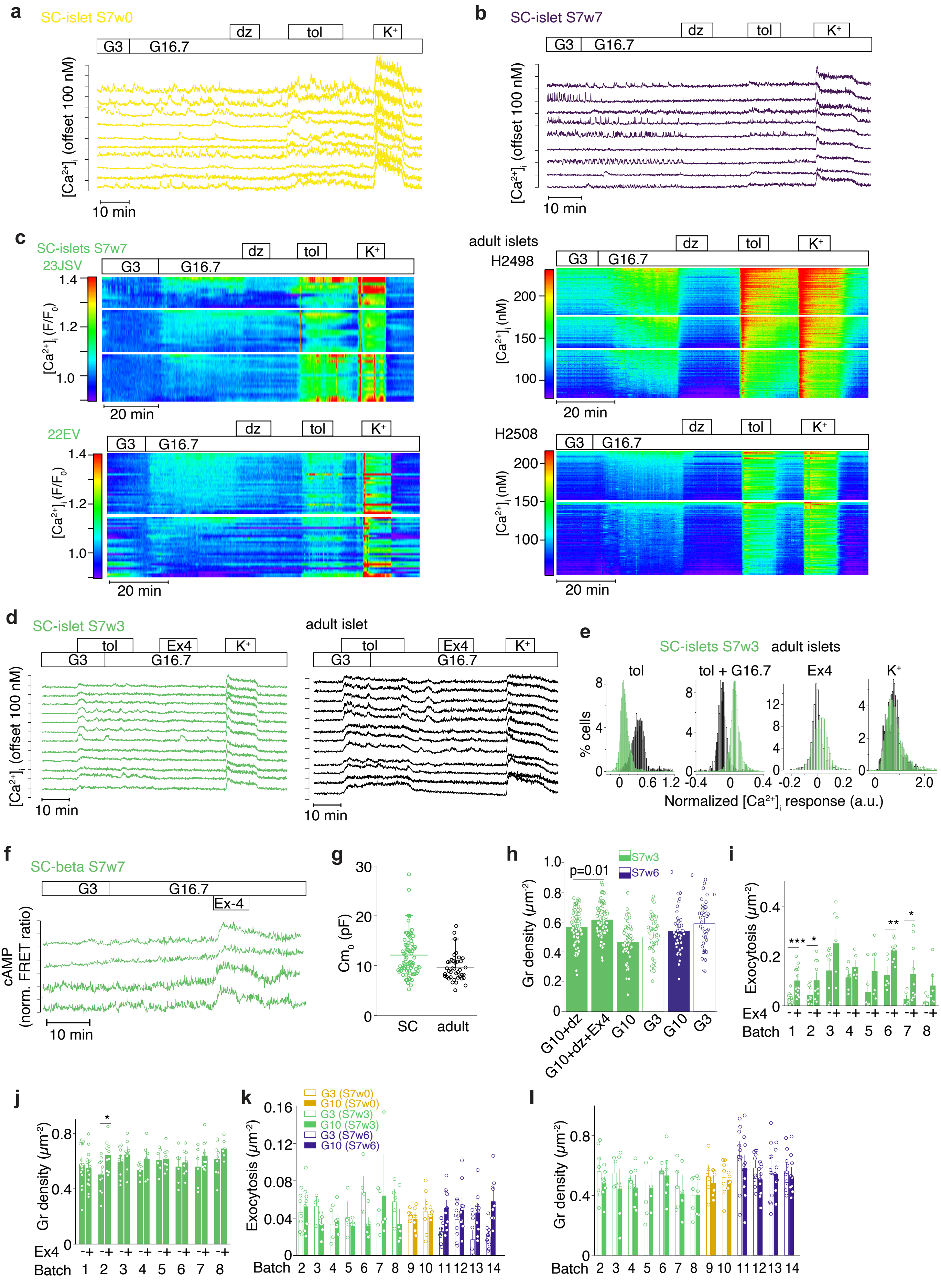

a SC-islet S7w0
b SC-islet S7w7
G3 G16.7 dz tol K+
[Ca2+]i (offset 100 nM)
10 min
c SC-islets S7w7
23JSV
22EV
adult islets
H2498
H2508
[Ca2+]i (F/F0)
[Ca2+]i (nM)
20 min
d SC-islet S7w3
adult islet
Ex4
e SC-islets S7w3 adult islets
tol
tol + G16.7
% cells
Normalized [Ca2+]i response (a.u.)
f SC-beta S7w7
Ex-4
cAMP (norm. FRET ratio)
g
Cm0 (pF)
SC adult
h
p=0.01
S7w3
S7w6
Gr density (μm-2)
G10+dz G10+dz+Ex4 G10 G3 G10 G3
i
Exocytosis (μm-2)
Ex4
Batch
j
k
G3 (S7w0)
G10 (S7w0)
G3 (S7w3)
G10 (S7w3)
G3 (S7w6)
G10 (S7w6)
l

Supplementary Figure 2

(a-b) Representative $[Ca^{2+}]i$ recordings from SC-islets at S7w0 (a) and S7w7 (b) during exposure to 3 (G3) and 16.7 mM (G16.7) glucose, 250 µM diazoxide, 1 mM tolbutamide and 30 mM K+.

(c) $[Ca^{2+}]i$ recordings under similar conditions as in (a), exemplifying inter-islet and inter-preparation variability in SC- and primary islets. Top left heatmap shows all R-GECO1-expressing SC-beta cells in three different SC-islets from one SC preparation (23JSV). Bottom left is a similar recording from two SC-islets from an independent SC-preparation (22EV). The heatmaps on the right show $[Ca^{2+}]i$ recordings with Fura-2 LR from three (top) and two (bottom) primary islets from two different human donors (H2498, H2508).

(d) $[Ca^{2+}]i$ recordings from SC-islets at S7w2 and primary islets exposed to 1 mM tolbutamide, G16.7, 10 nM exendin-4 and 30 mM K+.

(e) Histograms showing the changes of $[Ca^{2+}]i$ in response to various treatments from SC-islets (n=4079 cells) and primary islets (n=1187 cells).

(f) [cAMP]m recording from identified SC-beta cells exposed to G3, G16.7 and 10 nM Ex4.

(g) Whole cell capacitance as measure of cells size in SC- and primary islet cells. Symbols represent individual cells, and symbol color the cell preparation or donor.

(h) Docked granules measured as average granule density, normalized to footprint area, in conditions as indicated. Dots represent individual cells. $p = 0.01$, two-tailed Student's t-test.

(i) Total exocytosis during 40 s of K+-stimulation in different SC-islet preparations. Dots represent individual cells. Two-tailed Student's t-test.

(j) Average granule density normalized to the footprint area of different SC-islet preparations. Dots represent individual cells. Two-tailed Student's t-test.

(k) Total exocytosis during 3 minutes of incubation in different concentration of glucose for different SC-islet preparation. Dots represent individual cells. Two-tailed Student's t-test.

(l) Average granule density normalized to the footprint area of different batch/preparation of cells as indicated. Dots represent individual cells.

All data presented as means ± SEM unless otherwise indicated.

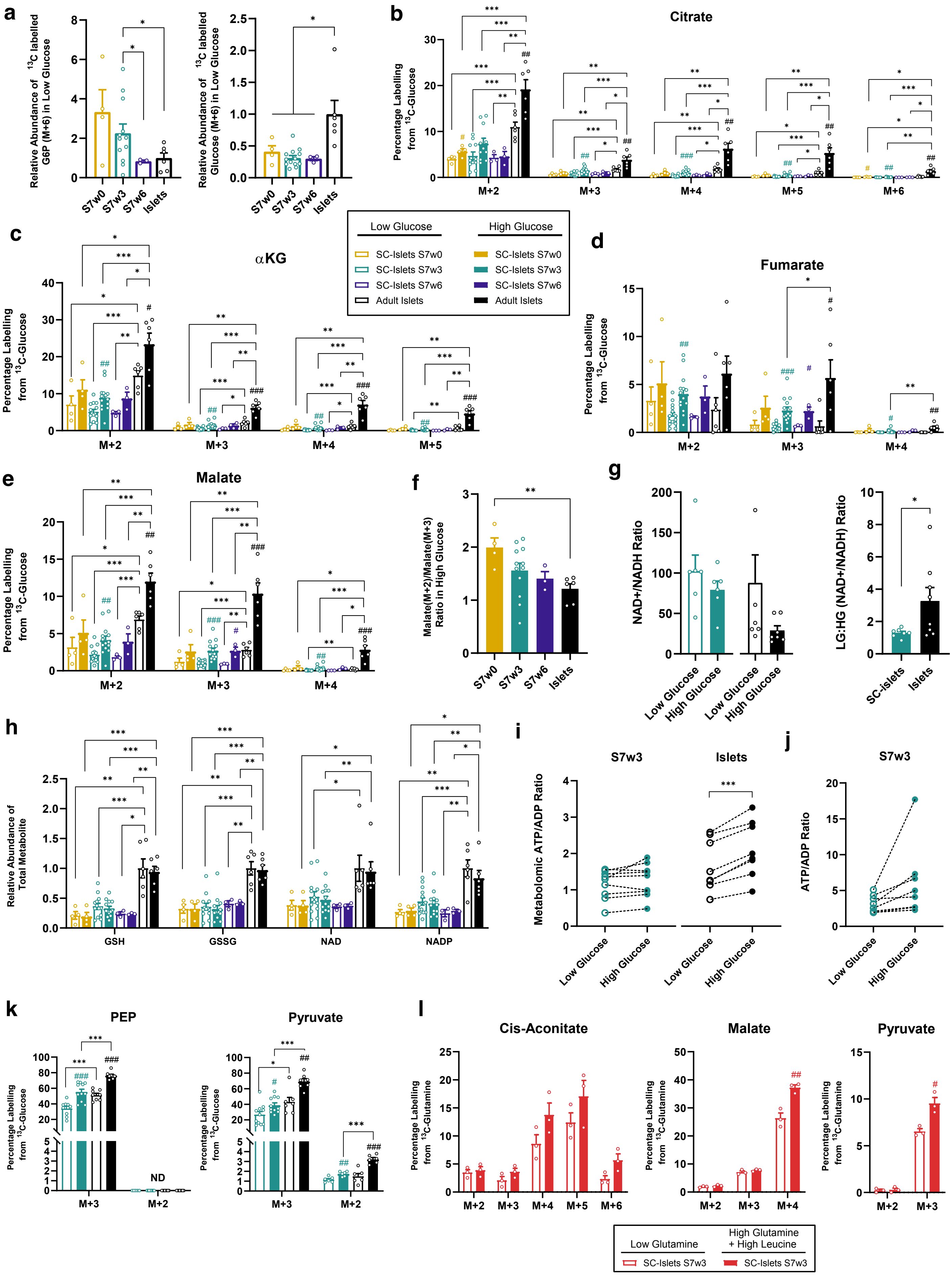

a
Relative Abundance of ^{13}C labelled G6P (M+6) in Low Glucose
Relative Abundance of ^{13}C labelled Glucose (M+6) in Low Glucose
S7w0
S7w3
S7w6
Islets
b
Citrate
Percentage Labelling from ^{13}C-Glucose
M+2
M+3
M+4
M+5
M+6
Low Glucose
High Glucose
SC-Islets S7w0
SC-Islets S7w3
SC-Islets S7w6
Adult Islets
c
αKG
Percentage Labelling from ^{13}C-Glucose
d
Fumarate
e
Malate
f
Malate(M+2)/Malate(M+3) Ratio in High Glucose
g
NAD+/NADH Ratio
LG:HG (NAD+/NADH) Ratio
Low Glucose
High Glucose
SC-Islets
Islets
h
Relative Abundance of Total Metabolite
GSH
GSSG
NAD
NADP
i
S7w3
Islets
Metabolomic ATP/ADP Ratio
j
S7w3
ATP/ADP Ratio
k
PEP
Pyruvate
Percentage Labelling from ^{13}C-Glucose
ND
l
Cis-Aconitate
Malate
Pyruvate
Percentage Labelling from ^{13}C-Glutamine
Low Glutamine
High Glutamine + High Leucine
SC-Islets S7w3

Supplementary Figure 3

(a) The abundance of M+6 labelled Glucose-6-Phosphate (G6P) and M+6 labelled Glucose retained under low glucose treatment of SC-islets from week 0 to week 6 of maturation, relative to adult islets.

(b-e), The percentage of total metabolite labelling following low and high glucose exposure for each isotopologue of the TCA metabolites citrate (b), alpha-ketoglutarate (αKG) (c), fumarate (d), and malate (e).

(f) The ratio of M+2 and M+3 labelled malate under high glucose treatment in SC-islets and adult islets.

(g) Metabolic data measurements of NAD+/NADH ratios following low and high glucose treatment in S7w3 SC-islets and adult islets (*left panel*). A ratio of the low glucose to high glucose shift of NAD+/NADH ratio in S7w3 SC-islets and adult islets (*right panel*).

(h), The combined total abundance of reduced and oxidized forms of glutathione (GSH and GSSG) and the reducing agents NAD and NADP in SC-islets and adult islets.

(i) Metabolic data measurements of ATP/ADP ratios in low and high glucose conditions in S7w3 SC-islets and adult islets.

(j) ATP/ADP ratio measurements under high and low glucose treatment in S7w3 SC-islets.

(k) Labelled enrichment of M+2 and M+3 isotopologues of PEP and pyruvate under low and high glucose treatment in S7w3 SC-islets and adult islets. ND = not detected.

(l) ^{13}C5-glutamine labelling of S7w3 SC-islets under low (2mM ^{13}C-glutamine) and high (10mM ^{13}C-glutamine with 5mM leucine) conditions and resulting isotopologue patterns of cis-aconitate, malate, and pyruvate.

Error bars ±SEM with statistical significance determined by two-tailed t-tests. '#' symbols indicate internal significance from low to high glucose labelling, '*' symbols denote significance between SC-islet timepoints or adult islet samples at each glucose concentration. $^{\#,*}p < 0.05$, $^{\#\#,**}p < 0.01$, $^{\#\#\#,***}p < 0.001$. SC-islets S7w0 (n=4), S7w3 (n=6-13), S7w6 (n=3), adult islets (n=6). For glutamine labelling experiments S7w3 (n=3).

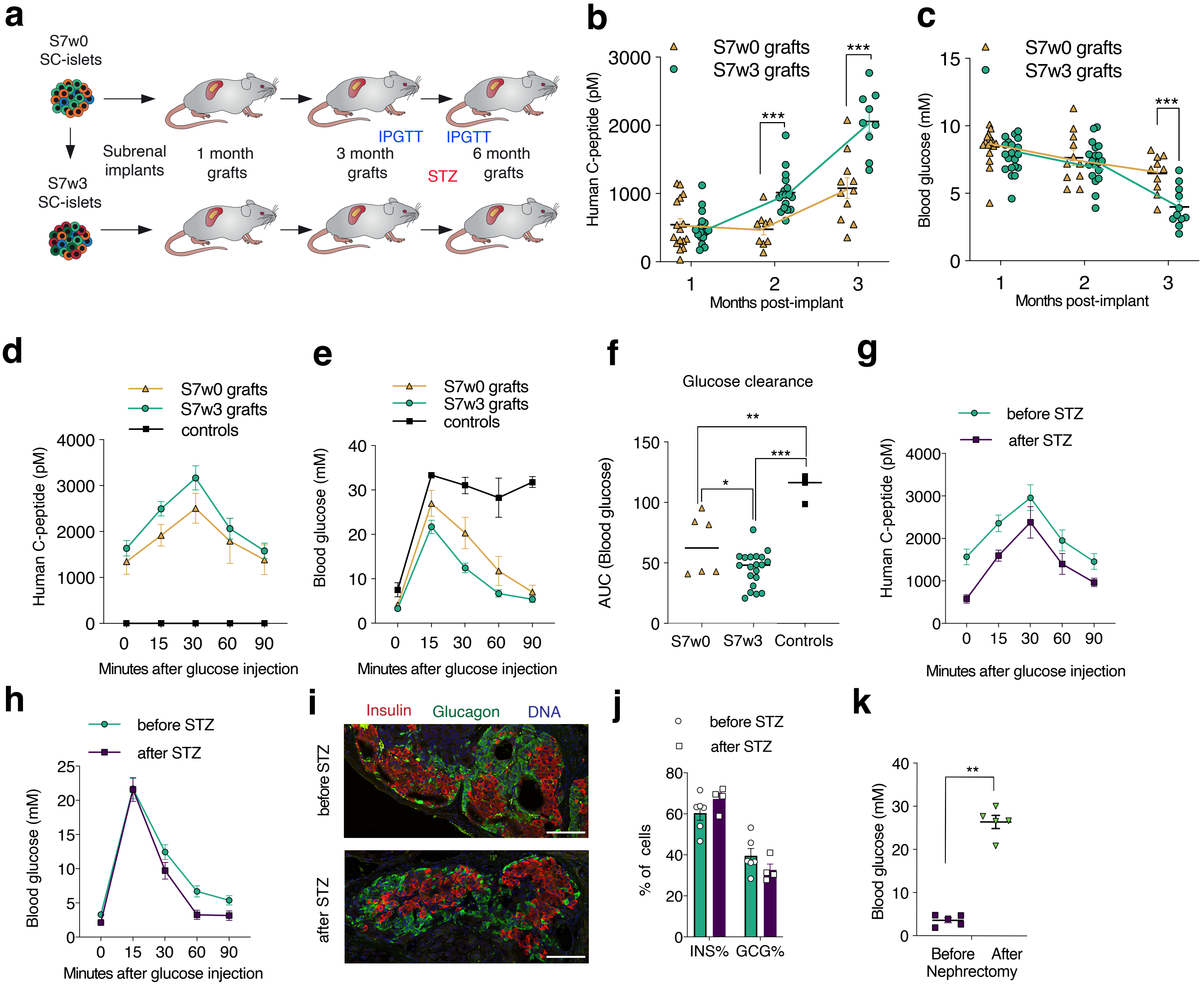

a
S7w0 SC-islets
Subrenal implants
1 month grafts
3 month grafts
IPGTT
IPGTT
STZ
6 month grafts
S7w3 SC-islets
b
S7w0 grafts
S7w3 grafts
Human C-peptide (pM)
Months post-implant
c
Blood glucose (mM)
Months post-implant
d
S7w0 grafts
S7w3 grafts
controls
Human C-peptide (pM)
Minutes after glucose injection
e
Blood glucose (mM)
Minutes after glucose injection
f
Glucose clearance
AUC (Blood glucose)
S7w0
S7w3
Controls
g
before STZ
after STZ
Human C-peptide (pM)
Minutes after glucose injection
h
before STZ
after STZ
Blood glucose (mM)
Minutes after glucose injection
i
Insulin
Glucagon
DNA
before STZ
after STZ
j
before STZ
after STZ
% of cells
INS%
GCG%
k
Blood glucose (mM)
Before
After
Nephrectomy

Supplementary Figure 4: Engrafted SC-islets provide effective glycemic control in mice

(a) Schematic representation of the *in vivo* experiments

(b-c) Measurements form random-fed mice engrafted with S7w0 or S7w3 SC-islets, **(b)** Human specific C-peptide levels (pM) and **(c)** Mouse blood glucose levels (mM) (S7w0: n=16 in 1m, 9-11 in 2m and 10-11 in 3m; S7w3: n=18-20 in 1m, 17-19 in 2m and 9-11 in 3m). Individual data points presented, Multiple two-tailed t-tests.

(d-e) Human C-peptide **(c)** and blood glucose **(d)** levels measured during an intraperitoneal glucose tolerance test (IPGTT) at three months post-engraftment. Mice were injected with 3 mg/kg glucose after a 6 h fast. (S7w0: n=6, S7w3: n=20, non-implanted controls n=3).

(f) Glucose clearance quantified from IPGTT blood glucose values in (d) as area under the curve (AUC). One-way ANOVA and Tukey's multiple comparisons test.

(g-h), (h-i) Human C-peptide levels (pM) **(g)** and blood glucose levels (mM) **(h)** in an IPGTT similar to the one in (d-e) in mice at 4 months post-engraftment (before streptozotocin (STZ) injection, n=10) and at 5 months post-engraftment (after STZ injection, n=9)

(i) Representative immunohistochemistry in the grafts before (n=6) and after (n=4) STZ injection. Scale bar 100µm.

(j) Quantification of INS^+ and GCG^+ populations before (n=6) and after (n=4) STZ injection, % of all cells positive for either INS or GCG. Dots represent graft-wide data from individual grafts.

(k) Blood glucose levels from engrafted, random-fed mice before and after nephrectomy at 5-6 months post-engraftment. The mice were injected with streptozotocin (STZ) at 4 months post-engraftment. (n=4) Two-sided Mann-Whitney U-test.

All data represent mean ±SEM, unless indicated otherwise * $p < 0.05$, ** $p < 0.01$, *** $p < 0.001$.

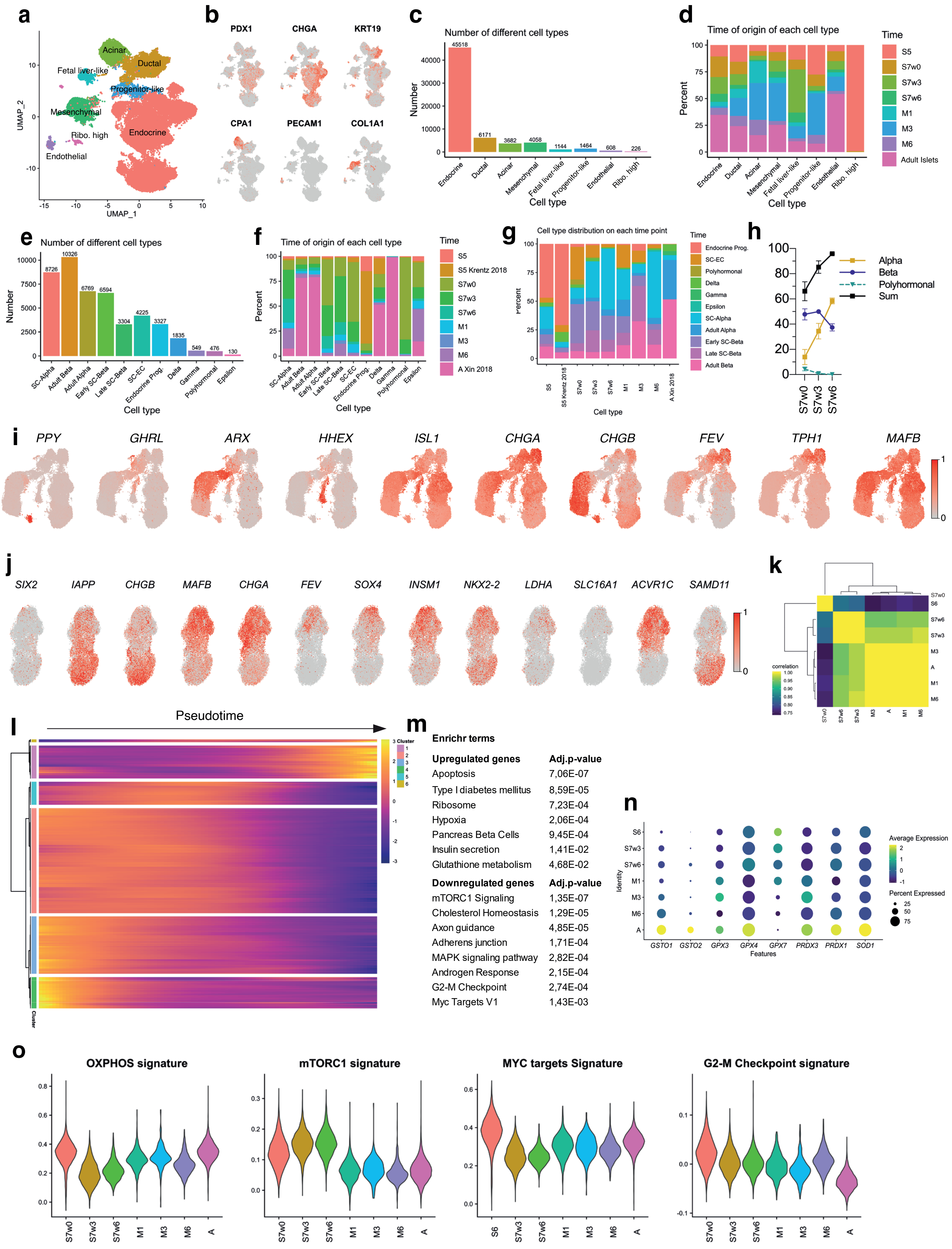
a
Acinar
Ductal
Fetal liver-like
Progenitor-like
Mesenchymal
Ribo. high
Endocrine
Endothelial
UMAP_1
UMAP_2
b
PDX1
CHGA
KRT19
CPA1
PECAM1
COL1A1
c
Number of different cell types
Number
Cell type
45518
6171
3682
4058
1144
1464
608
226
Endocrine
Ductal
Acinar
Mesenchymal
Fetal liver-like
Progenitor-like
Endothelial
Ribo. high
d
Time of origin of each cell type
Percent
Cell type
Time
S5
S7w0
S7w3
S7w6
M1
M3
M6
Adult Islets
e
Number of different cell types
Number
Cell type
8726
10326
6769
6594
3304
4225
3327
1835
549
476
130
SC-Alpha
Adult Beta
Adult Alpha
Early SC-Beta
Late SC-Beta
SC-EC
Endocrine Prog.
Delta
Gamma
Polyhormonal
Epsilon
f
Time of origin of each cell type
Percent
Cell type
Time
S5
S5 Krentz 2018
S7w0
S7w3
S7w6
M1
M3
M6
A Xin 2018
g
Cell type distribution on each time point
Percent
Cell type
Time
Endocrine Prog.
SC-EC
Polyhormonal
Delta
Gamma
Epsilon
SC-Alpha
Adult Alpha
Early SC-Beta
Late SC-Beta
Adult Beta
h
Alpha
Beta
Polyhormonal
Sum
S7w0
S7w3
S7w6
i
PPY
GHRL
ARX
HHEX
ISL1
CHGA
CHGB
FEV
TPH1
MAFB
j
SIX2
IAPP
CHGB
MAFB
CHGA
FEV
SOX4
INSM1
NKX2-2
LDHA
SLC16A1
ACVR1C
SAMD11
k
correlation
S7w0
S6
S7w6
S7w3
M3
A
M1
M6
l
Pseudotime
Cluster
m
Enrichr terms
Upregulated genes	Adj.p-value
Apoptosis	7,06E-07
Type I diabetes mellitus	8,59E-05
Ribosome	7,23E-04
Hypoxia	2,06E-04
Pancreas Beta Cells	9,45E-04
Insulin secretion	1,41E-02
Glutathione metabolism	4,68E-02
Downregulated genes	Adj.p-value
mTORC1 Signaling	1,35E-07
Cholesterol Homeostasis	1,29E-05
Axon guidance	4,85E-05
Adherens junction	1,71E-04
MAPK signaling pathway	2,82E-04
Androgen Response	2,15E-04
G2-M Checkpoint	2,74E-04
Myc Targets V1	1,43E-03
n
Identity
Features
Average Expression
Percent Expressed
GSTO1
GSTO2
GPX3
GPX4
GPX7
PRDX3
PRDX1
SOD1
o
OXPHOS signature
mTORC1 signature
MYC targets Signature
G2-M Checkpoint signature
S7w0
S6
S7w3
S7w6
M1
M3
M6
A

Supplementary Figure 5

(a) UMAP projection of the full integrated scRNAseq dataset, containing 62 871 cells, colored by different cell types.

(b) Relative expression of marker genes for pancreatic (*PDX1*), endocrine (*CHGA*), ductal (*KRT19*), acinar (*CPA1*), endothelial (*PECAM1*) and mesenchymal (*COL1A1*) cells.

(c) Numbers of each different cell type in the full integrated scRNAseq dataset.

(d) Fractional contribution of each timepoint to each cell type identity in the full integrated scRNAseq dataset.

(e) Numbers of each different cell type in the filtered endocrine scRNAseq dataset.

(f) Fractional contribution of each timepoint to each cell type identity in the filtered endocrine dataset.

(g) Fractional contribution of each cell type identity to each timepoint in the filtered endocrine dataset

(h) Percentage of cells in the alpha, beta and polyhormonal clusters and the sum of these clusters in the S7 culture timepoints. Data presented as means ± SEM.

(i) Relative expression of marker genes for the different endocrine cell type identities.

(j) Relative expression of beta cell marker genes in the beta cell subpopulations.

(k) Pearson correlation of the average gene expression in the cells from each different time point.

(l) Hierarchical clustering of the top 1500 genes most differentially regulated along pseudotime (see also Supp.Table.4).

(m) Gene sets enriched in the upregulated and downregulated genes along pseudotime presented in (Fig.5l) Pseudotime clusters with upregulated genes 1 and 6, and cluster with downregulated genes 2,3,4 and 5 were grouped for the gene set enrichment analysis.

(n) Expression of glutathione metabolism-related genes across beta cells from different time of origin.

(o) Signatures of processes differentially regulated along pseudotime on beta cells from each different time point.

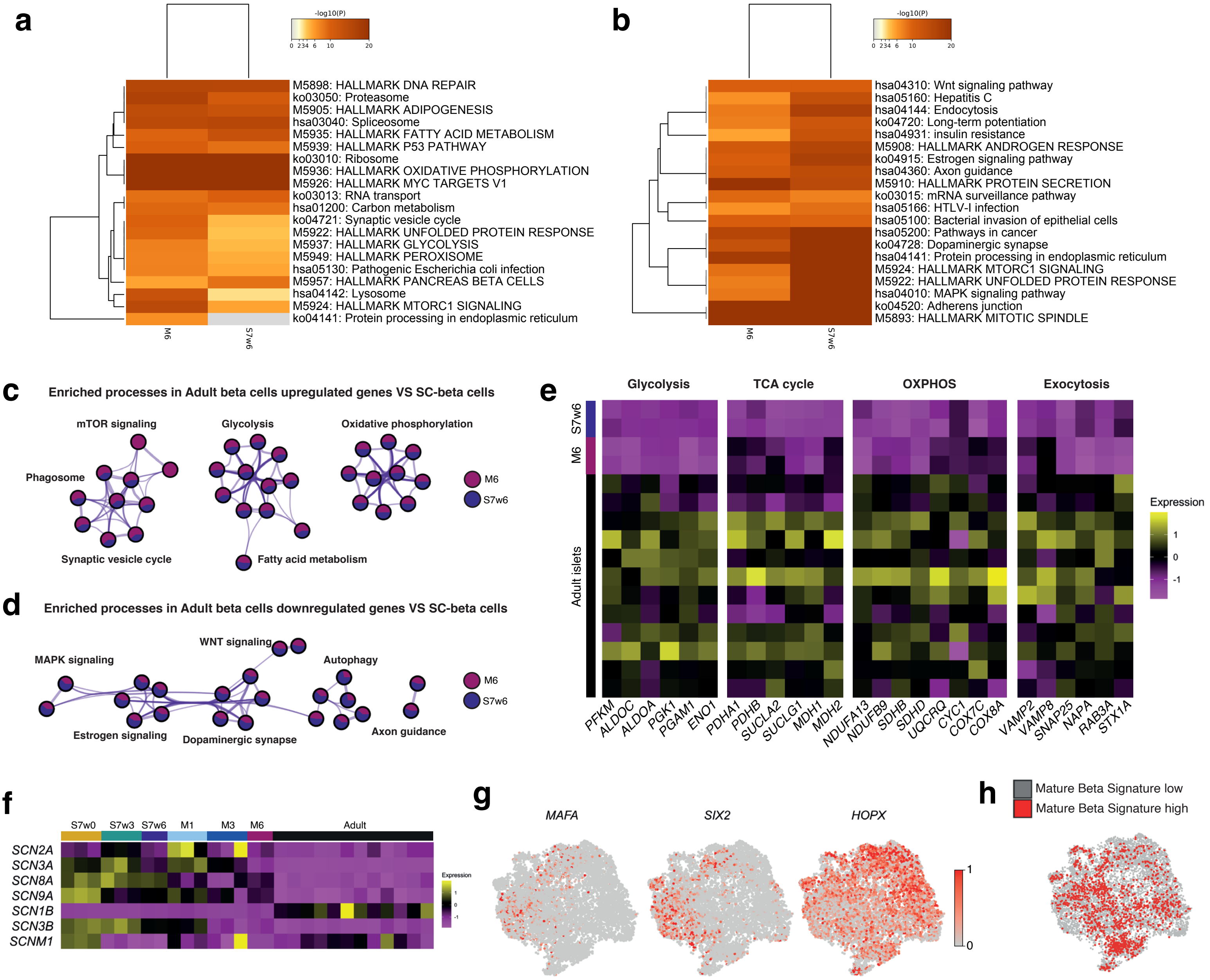

a
-log10(P)
0 2 3 4 6 10 20
M5898: HALLMARK DNA REPAIR
ko03050: Proteasome
M5905: HALLMARK ADIPOGENESIS
hsa03040: Spliceosome
M5935: HALLMARK FATTY ACID METABOLISM
M5939: HALLMARK P53 PATHWAY
ko03010: Ribosome
M5936: HALLMARK OXIDATIVE PHOSPHORYLATION
M5926: HALLMARK MYC TARGETS V1
ko03013: RNA transport
hsa01200: Carbon metabolism
ko04721: Synaptic vesicle cycle
M5922: HALLMARK UNFOLDED PROTEIN RESPONSE
M5937: HALLMARK GLYCOLYSIS
M5949: HALLMARK PEROXISOME
hsa05130: Pathogenic Escherichia coli infection
M5957: HALLMARK PANCREAS BETA CELLS
hsa04142: Lysosome
M5924: HALLMARK MTORC1 SIGNALING
ko04141: Protein processing in endoplasmic reticulum
M6
S7w6
b
-log10(P)
0 2 3 4 6 10 20
hsa04310: Wnt signaling pathway
hsa05160: Hepatitis C
hsa04144: Endocytosis
ko04720: Long-term potentiation
hsa04931: insulin resistance
M5908: HALLMARK ANDROGEN RESPONSE
ko04915: Estrogen signaling pathway
hsa04360: Axon guidance
M5910: HALLMARK PROTEIN SECRETION
ko03015: mRNA surveillance pathway
hsa05166: HTLV-I infection
hsa05100: Bacterial invasion of epithelial cells
hsa05200: Pathways in cancer
ko04728: Dopaminergic synapse
hsa04141: Protein processing in endoplasmic reticulum
M5924: HALLMARK MTORC1 SIGNALING
M5922: HALLMARK UNFOLDED PROTEIN RESPONSE
hsa04010: MAPK signaling pathway
ko04520: Adherens junction
M5893: HALLMARK MITOTIC SPINDLE
M6
S7w6
c
Enriched processes in Adult beta cells upregulated genes VS SC-beta cells
mTOR signaling
Glycolysis
Oxidative phosphorylation
Phagosome
Synaptic vesicle cycle
Fatty acid metabolism
M6
S7w6
d
Enriched processes in Adult beta cells downregulated genes VS SC-beta cells
WNT signaling
MAPK signaling
Autophagy
Estrogen signaling
Dopaminergic synapse
Axon guidance
M6
S7w6
e
Glycolysis
TCA cycle
OXPHOS
Exocytosis
S7w6
M6
Adult islets
Expression
1
0
-1
PFKM
ALDOC
ALDOA
PGK1
PGAM1
ENO1
PDHA1
PDHB
SUCLA2
SUCLG1
MDH1
MDH2
NDUFA13
NDUFB9
SDHB
SDHD
UQCRQ
CYC1
COX7C
COX8A
VAMP2
VAMP8
SNAP25
NAPA
RAB3A
STX1A
f
S7w0
S7w3
S7w6
M1
M3
M6
Adult
SCN2A
SCN3A
SCN8A
SCN9A
SCN1B
SCN3B
SCNM1
Expression
1
0
-1
g
MAFA
SIX2
HOPX
1
0
h
Mature Beta Signature low
Mature Beta Signature high

Supplementary Figure 6

(a-b) Gene sets enriched in the upregulated (a) and downregulated (b) genes primary adult beta cells compared to S7w6 *in vitro* and M6 *in vivo* SC-beta cells.

(c-d) Network of enriched biological pathways showing the relationships among processes that are upregulated (c) or downregulated (d) in primary adult beta cells compared to S7w6 *in vitro* and M6 *in vivo* SC-beta cells.

Enriched biological pathways can be represented in a network, which facilitates the understanding of relationships among biological pathways or processes,

(e) Expression of genes that are significantly upregulated in primary adult beta cells compared to S7w6 *in vitro* and M6 *in vivo* SC-beta cells, averaged by individual sample.

(f) Average expression of sodium channel subunit genes across beta cells from different time of origin.

(g) UMAP projection of in vitro SC-beta cells annotated by the heterogeneous expression of mature beta cell markers.

(h) UMAP projection of in vitro SC-beta cells annotated by high or low mature beta signature score.

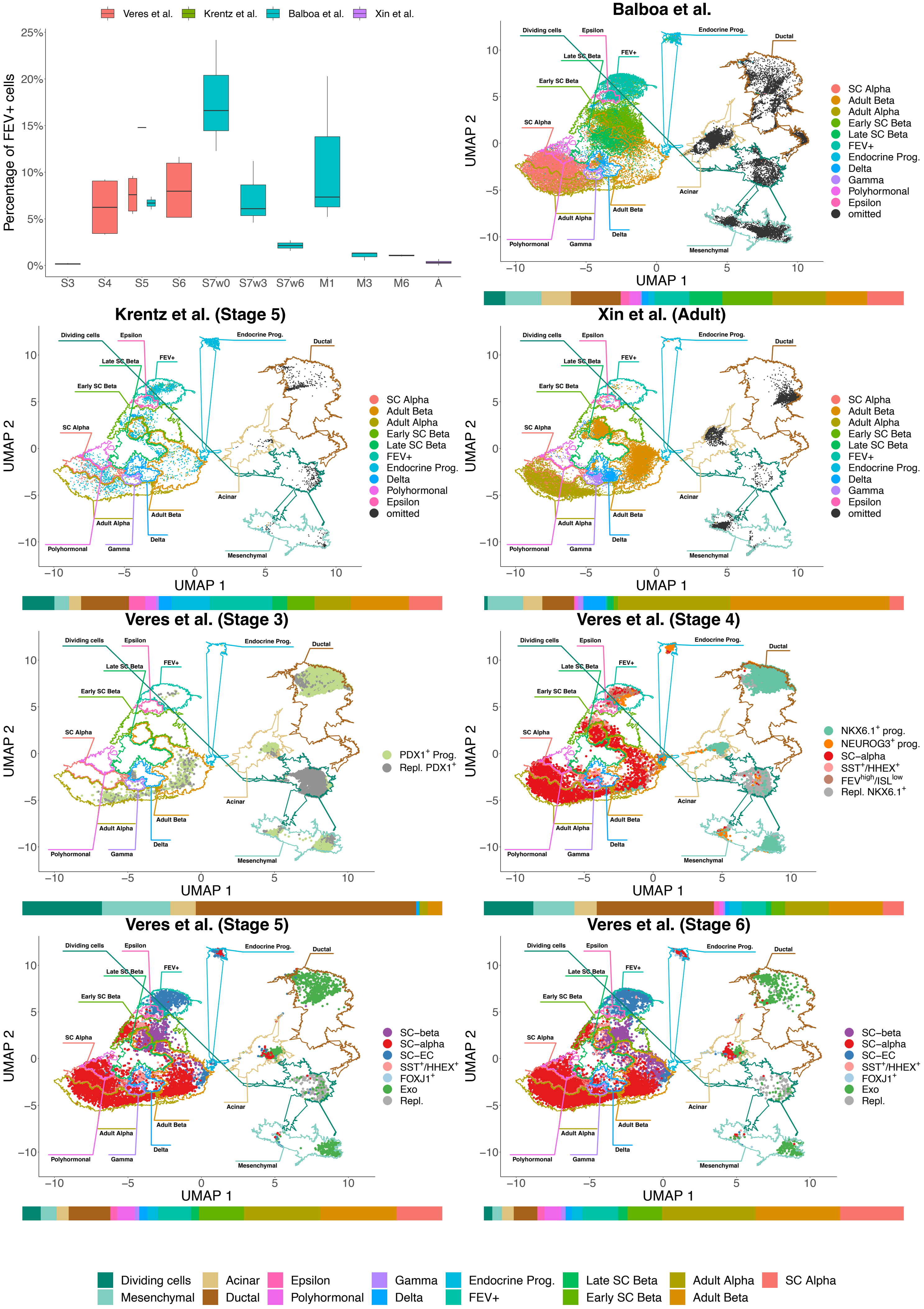
Veres et al.
Krentz et al.
Balboa et al.
Xin et al.
Percentage of FEV+ cells
S3
S4
S5
S6
S7w0
S7w3
S7w6
M1
M3
M6
A
Balboa et al.
Krentz et al. (Stage 5)
Xin et al. (Adult)
Veres et al. (Stage 3)
Veres et al. (Stage 4)
Veres et al. (Stage 5)
Veres et al. (Stage 6)
UMAP 1
UMAP 2
SC Alpha
Adult Beta
Adult Alpha
Early SC Beta
Late SC Beta
FEV+
Endocrine Prog.
Delta
Gamma
Polyhormonal
Epsilon
omitted
PDX1+ Prog.
Repl. PDX1+
NKX6.1+ prog.
NEUROG3+ prog.
SC−alpha
SST+/HHEX+
FEVhigh/ISLlow
Repl. NKX6.1+
SC−beta
SC−EC
FOXJ1+
Exo
Repl.
Dividing cells
Mesenchymal
Acinar
Ductal

Supplementary Figure 7

(a) Percentage of SC-EC cells detected in the different time points of each scRNAseq datasets included in the comparison (Veres et al., Krentz et al., Xin et al, and this study). Boxplots represent interquartile range (IQR) showing the median, the 75th and the 25th percentile. The whiskers denote roughly 95% of the data range (1.5*IQR).

(b-h) UMAP projections indicating the similar clustering of equivalent cell types arising from the different scRNAseq datasets included in the comparison (Veres et al., Krentz et al., Xin et al, and this study). Similar cell types are coloured and labelled according to our cell type annotations. The proportions of cell types for each study are shown with bar graphs with corresponding colours for each cell type.

Supplementary table 9: differentiation medium composition

Stage	Duration (d)	Culture format	Basal medium	Additives
1	3	Matrigel coated planar	#1: MCDB131 (10372-019, Life Technologies)<br>+ 2 mM Glutmax (35050038, Life Technologies)<br>+ 1.5 g/l NaHCO3 (Sigma-Aldrich)<br>+ 5 g/l BSA fV (Sigma-Aldrich, A7030)<br>+ 5 mM glucose.<br>Total glucose 10.44 mM	+ 100 ng/ml Activin A (QKine limited, UK)<br>+ CHIR (Tocris, #4423) 3.0 µM on the 1st day, 0.3 µM on the 2nd day, 0.0 µM on the 3rd day.
2	3	Matrigel coated planar		+ 50 ng/ml FGF7 (Genscript, Z03047)<br>+ 0.25 mM Vitamin C (Sigma, A4544)
3	2	Matrigel coated planar	#2: MCDB131<br>+ 2 mM Glutmax<br>+ 2.5 g/l NaHCO3<br>+ 20 g/l BSA fV<br>+ 5 mM glucose<br>+ 0.5% ITS-X (supp).<br>Total glucose 10.42 mM	+ 50 ng/ml FGF7<br>+ 0.25 mM Vitamin C<br>+ 0.25 µM SANT1 (Sigma, S4572)<br>+ 1 µM Retinoic Acid (Sigma, R2625)<br>+ 100 nM LDN (Selleckchem, S2618)<br>+ 200 nM TPB (Santa Cruz, Sc-204424)
4	4	Planar -> Aggre Well 400		+ 10 mM Nicotinamide (N0636, SIGMA)<br>+0.25 mM Vitamin C<br>+ 100 ng/ml EGF (AF-100-15, Peprotech)<br>+ 10 µM ROCKi<br>+ 2 ng/ml FGF7<br>+ 0.25 µM SANT1<br>+ 0.1 µM Retinoic Acid<br>+ 200 nM LDN<br>+ 100 nM TPB<br>+ 10 ng/ml Activin A
5	4	Aggre Well 400	#3: MCDB131<br>+ 2 mM Glutmax<br>+ 1.5 g/l NaHCO3<br>+ 20 g/l BSA fV<br>+ 15 mM glucose<br>+ 0.5% ITS-X<br>+ 10 µg/ml Heparin (H3149, Sigma-Aldrich)<br>+ 10 µM $ZnSO_4$ (Z0251, SigmaAldrich)<br>+ 1% Penicillin-Streptomycin.<br>Total glucose 20.13 mM	+ 0.25 µM SANT1<br>+ 0.05 µM retinoic acid<br>+100 nM LDN<br>+ 10 µM ALK5inhII (S7233, Selleckchem)<br>+ 1 µM GC1 (4554, Tocris)<br>+ 20 ng/mL Betacellulin (100-50, Peprotech)<br>+ 100 nM GSiXX (565789, Millipore)
6	7-8	Suspension		+ 100 nM LDN<br>+ 10 µM ALK5inhII<br>+ 1 µM GC1<br>+ 100 nM GSiXX
7	0-42	Suspension	CMRL1066 (15-110-CVR, Corning)<br>+ 2 mM Glutmax<br>+ 20 g/l BSA fV<br>+ 0.5% ITS-X<br>+ 10 µg/l heparin<br>+ 10 µg/l $ZnSO_4$<br>+ 0.5 mM Sodium Pyruvate (Lonza, BE13-115E)<br>+ 1:2000 Trace elements A (25-021-CI, Cellgro)<br>+ 1:2000 Trace elements B (99-175-CI, Cellgro)<br>+ 1:2000 Lipid concentrate (11905-031, Invitrogen)<br>+ 1% Penicillin-Streptomycin.<br>Total glucose 5.32 mM	+ 0.5 µM ZM447439 (ZM, Selleckchem, S1103)<br>+ 10 nM Tri-iodothyronine (T3) +<br>+ 1 mM N-Acetyl-Cysteine (NAC, A9165, Sigma-Aldrich)

Supplementary table 10: Human islet characteristics

Shipment#	Source	Purity (%)	Age (y)	Sex	BMI (kg/m^2)	HbA1c (mmol/mol)
1	Uppsala	94	67	M	26.1	38
2	Uppsala	97	65	M	30.1	42
3	Uppsala	96	71	M	22.4	41
4	Uppsala	72	36	F	18.9	32
5	Uppsala	98	61	F	24.7	35
6	Uppsala	80	70	F	22.0	32
7	Alberta R389	75	65	F	24.4	37
8	Uppsala	69	51	F	28.1	35
9	Uppsala	86	51	M	24.8	37
10	Uppsala	96	54	M	41.6	39

Uppsala = Nordic Network for Islet Transplantation, Uppsala University, Sweden
Alberta = IsletCore, University of Alberta, Canada

Supplementary table 11: antibodies used

**Epitope**	**Origin animal**	**Conjugate**	**Dilution**	**Supplier**	**Assay**
PDX1	Mouse	PE	1:80	BD Biosciences #562161	FC
NKX6.1	Mouse	Red 647	1:80	BD Biosciences #563338	FC
Insulin	Rabbit	Red 647	1:160	Cell Signaling Technology Cat# 9008	FC
Insulin	Guinea pig	N/A	1:500	DAKO, A0564	IHC
Glucagon	Mouse	N/A	1:160/ 1:500	Sigma-Aldrich, #G2654	FC / IHC
Somatostatin	Rabbit	N/A	1:500	DAKO, A0566	IHC
Ki-67	Rabbit	N/A	1:500	Leica Microsystems #NCL-Ki67p	IHC
SLC18A1	Rabbit	N/A	1:500	Sigma-Aldrich, #HPA063797	IHC
LDHA	Rabbit	N/A	1:250	Cell Signaling Technology Cat#3582	IHC
Guinea pig	Goat	Red 594	1:500	Thermo-Fisher, #11076	IHC
Mouse	Donkey	Green 488	1:500	Thermo-Fisher, #21202	FC/IHC
Rabbit	Donkey	Green 488	1:500	Thermo-Fisher, #21206	IHC

FC= Flow cytometry, IHC=Immunohistochemisty
