# Supplementary material for: Functional, metabolic and transcriptional maturation of human pancreatic islets derived from stem cells
Source: Nat Biotechnol. 2022 Mar 3;40(7):1042–55. doi: 10.1038/s41587-022-01219-z (PMC9287162; doi:10.1038/s41587-022-01219-z)
Supplement: Supplementary file 2 — Reporting Summary. [file 41587_2022_1219_MOESM2_ESM.pdf]

nature research

Corresponding author(s): Timo Otonkoski

Last updated by author(s): Dec 22, 2021

# Reporting Summary

Nature Research wishes to improve the reproducibility of the work that we publish. This form provides structure for consistency and transparency in reporting. For further information on Nature Research policies, see our Editorial Policies and the Editorial Policy Checklist.

## Statistics

For all statistical analyses, confirm that the following items are present in the figure legend, table legend, main text, or Methods section.

| n/a | Confirmed | |
|---|---|---|
| ☐ | ☒ | The exact sample size ($n$) for each experimental group/condition, given as a discrete number and unit of measurement |
| ☐ | ☒ | A statement on whether measurements were taken from distinct samples or whether the same sample was measured repeatedly |
| ☐ | ☒ | The statistical test(s) used AND whether they are one- or two-sided<br>*Only common tests should be described solely by name; describe more complex techniques in the Methods section.* |
| ☒ | ☐ | A description of all covariates tested |
| ☐ | ☒ | A description of any assumptions or corrections, such as tests of normality and adjustment for multiple comparisons |
| ☐ | ☒ | A full description of the statistical parameters including central tendency (e.g. means) or other basic estimates (e.g. regression coefficient) AND variation (e.g. standard deviation) or associated estimates of uncertainty (e.g. confidence intervals) |
| ☐ | ☒ | For null hypothesis testing, the test statistic (e.g. $F$, $t$, $r$) with confidence intervals, effect sizes, degrees of freedom and $P$ value noted<br>*Give P values as exact values whenever suitable.* |
| ☒ | ☐ | For Bayesian analysis, information on the choice of priors and Markov chain Monte Carlo settings |
| ☒ | ☐ | For hierarchical and complex designs, identification of the appropriate level for tests and full reporting of outcomes |
| ☐ | ☒ | Estimates of effect sizes (e.g. Cohen's $d$, Pearson's $r$), indicating how they were calculated |

*Our web collection on statistics for biologists contains articles on many of the points above.*

## Software and code

Policy information about availability of computer code

| | |
|---|---|
| Data collection | BD Cellquest Pro v4.0.2, Axio Scan Blue Zeiss software Zen2 Blue Edition v2, Tracefinder v4.1 |
| Data analysis | GraphPad Prism 8.4.2, CellProfiler 4.0, FlowJo v10, Tracefinder 4.1, 10x Genomics Cell Ranger v3.1, DropletUtils v1.6.1, Seurat v3.2.3, Harmony v1.0, Monocle2 v2.14.0, Soupx v1.4.8, Rmagic v2.0.3, velocyto v0.17.17, scVelo v0.2.2, scanpy v1.5.0, Enrichr, CellPhoneDB v2.0.0, Patchmaster v2x90, MetaFluor 7.7, Igor Pro 8, IsoCor plugin for Python, Agilent Wave v2.6 |

For manuscripts utilizing custom algorithms or software that are central to the research but not yet described in published literature, software must be made available to editors and reviewers. We strongly encourage code deposition in a community repository (e.g. GitHub). See the Nature Research guidelines for submitting code & software for further information.

## Data

Policy information about availability of data

All manuscripts must include a data availability statement. This statement should provide the following information, where applicable:

- Accession codes, unique identifiers, or web links for publicly available datasets
- A list of figures that have associated raw data
- A description of any restrictions on data availability

Single cell RNA-seq data were deposited to GEO under accession number GSE167880, which is publicly accessible. All other data are available from the authors

# Field-specific reporting

Please select the one below that is the best fit for your research. If you are not sure, read the appropriate sections before making your selection.

☒ Life sciences ☐ Behavioural & social sciences ☐ Ecological, evolutionary & environmental sciences

For a reference copy of the document with all sections, see nature.com/documents/nr-reporting-summary-flat.pdf

# Life sciences study design

All studies must disclose on these points even when the disclosure is negative.

| | |
|---|---|
| Sample size | No statistical method was used to determine sample size. Instead, biological repeats were collected based on availability of primary tissue. Stem cell derived tissue was tested with adequate repeats to confirm consistent data between parallel experimental setups. The range of biological repeats was therefore between n=3 to n=18 depending on availability of material and inherent variability within each assay. |
| Data exclusions | Low functioning primary islets (through batch variation or low cell viability) were excluded from the GSIS data sets. Metabolite tracing excluded peak data for low detection/non-specific metabolite measurements during data analysis. scRNAseq datasets excluded cell data with low reads or markers of high mitochondrial stress. |
| Replication | SC-islet differentiations were carried out by multiple researchers utilising the same protocol, and all produced SC-islet batches with similar levels of functionality and measurements between the multiple assays within the study. The number of independent SC-islet experiments used for each assay is detailed in the figure legends and up to 18 independent experiments with similar results were used in some of the functional tests. No SC-islet experiments were excluded from analysis, unless a technical issue with data collection occurred. |
| Randomization | No interventions requiring randomization were used for the study. When choosing SC-islets for various analyses, the samples were collected randomly from the whole batch. |
| Blinding | IHC immunostaining and quantification was blinded through processing batches simultaneously in the CellProfiler pipeline. Due to the experimental nature of comparing stem cell derived tissue and sporadically available primary tissue samples, no blinding could be achieved during experimental data collection. Data analysis was performed in a simultaneous and unbiased manner for all collected samples, where no blinding was necessary. |

# Reporting for specific materials, systems and methods

We require information from authors about some types of materials, experimental systems and methods used in many studies. Here, indicate whether each material, system or method listed is relevant to your study. If you are not sure if a list item applies to your research, read the appropriate section before selecting a response.

## Materials & experimental systems

| n/a | Involved in the study | |
|---|---|---|
| ☐ | ☒ | Antibodies |
| ☐ | ☒ | Eukaryotic cell lines |
| ☒ | ☐ | Palaeontology and archaeology |
| ☐ | ☒ | Animals and other organisms |
| ☒ | ☐ | Human research participants |
| ☒ | ☐ | Clinical data |
| ☒ | ☐ | Dual use research of concern |

## Methods

| n/a | Involved in the study | |
|---|---|---|
| ☒ | ☐ | ChIP-seq |
| ☐ | ☒ | Flow cytometry |
| ☒ | ☐ | MRI-based neuroimaging |

## Antibodies

| | |
|---|---|
| Antibodies used | Anti-insulin, Alexa 647 conjugate (FC), Rabbit, 1:160, Cell signaling technology (Cat#9008)<br>Anti-insulin (IHC), Guinea pig, 1:500, DAKO A0564<br>Anti-glucagon (FC,IHC), Mouse, 1:160 (FC) / 1:500 (IHC), Sigma-Aldrich #G2654)<br>Anti-somatostatin (IHC), Rabbit, 1:500, DAKO A21206<br>Anti-Ki-67 (IHC), Rabbit, 1:500, Leica Microsystems #NCL-Ki67p<br>Anti-SLC18A1 (IHC), Rabbit, 1:500, Merck HPA063797<br>Anti-LDHA (IHC), Rabbit, 1:250, Cell Signaling Technology #3582<br>Anti-Guinea pig Alexa 594 conjugate (IHC), Goat, 1:500, Thermo-Fisher #A-11076<br>Anti-Mouse Alexa 488 conjugate (FC/IHC), Donkey, 1:500, Thermo-Fisher #A-21202<br>Anti-Rabbit Alexa 488 conjugate (IHC), Donkey, 1:500; Thermo-Fisher #A-21206 |
| Validation | All antibodies used were validated using primary islet tissue or in-house tissue samples. The anti-Insulin-AF647 antibody has flow cytometry validation data ("tested in-house for direct immunofluorescent analysis in rat cells and flow cytometry in mouse cells") and relevant citations on the Cell Signaling Technology website. The anti-insulin antibody (DAKO) has numerous citations of use for |

specific immunohistochemical staining. The glucagon antibody has IF validation images on the Sigma-Aldrich website as well as supplied relevant citations. The Ki-67 antibody has relevant immunohistochemical staining data on the product website (Leica). The SLC18A1 antibody has relevant IHC data on the product website (Merck) and is cited in recent publications (Veres et al.). The LDHA antibody has relevant IHC and citation data on the product website (CST). The anti-guinea pig AF594, anti-mouse AF488, and the anti-rabbit AF 488 antibodies all have numerous relevant citations of validated function on the product website (Thermo Fisher).

## Eukaryotic cell lines

Policy information about cell lines

| | |
|---|---|
| Cell line source(s) | H1 hESCs were purchased from WiCell. HEL24.3 and HEL113.5 iPSC lines were derived in Biomedicum Helsinki Stem Cell Core |
| Authentication | De novo iPSC lines pluripotency and genomic stability were validated by the BSCC core facility with mehods including G-band karyotyping, immunohistochemistry and quantitative PCR for pluripotency markers (OCT4, NANOG, TRA1-60, SOX2, SSEA3), teratoma assays and Promega StemElite STR ID system. HEL24.3 authenticated in Trokovic et. al 2015 and HEL113.5 in Lithovius et al. 2021 |
| Mycoplasma contamination | The hPSCs were tested routinely for Mycoplasma and they tested negative |
| Commonly misidentified lines (See ICLAC register) | not used |

## Animals and other organisms

Policy information about studies involving animals; ARRIVE guidelines recommended for reporting animal research

| | |
|---|---|
| Laboratory animals | Mus Musculus, NOD-Scid-Gamma, Male only, Age 2-10 months at SC-islet implantation. Mice maintained at the Biomedicum Helsinki animal facility on a 12-h light/dark cycle with ad libitum food. The temperature was kept at 23°C with 24 relative humidity (RH) |
| Wild animals | No wild animals were used in this study |
| Field-collected samples | No field collected samples were used in this study |
| Ethics oversight | Animal care and experiments were approved by National Animal Experiment Board in Finland (ESAVI/14852/2018) |

Note that full information on the approval of the study protocol must also be provided in the manuscript.

## Flow Cytometry

### Plots

Confirm that:

☒ The axis labels state the marker and fluorochrome used (e.g. CD4-FITC).

☒ The axis scales are clearly visible. Include numbers along axes only for bottom left plot of group (a 'group' is an analysis of identical markers).

☒ All plots are contour plots with outliers or pseudocolor plots.

☒ A numerical value for number of cells or percentage (with statistics) is provided.

### Methodology

| | |
|---|---|
| Sample preparation | SC-islets were dissociated with TryplE for 8-10 minutes before single-cell filtering and fixation/permeabilisation in BD Cytofix/CytoPerm solution for 20 minutes at room temperature. Primary antibodies were incubated overnight at 4C in a 5% FBS PBS solution, secondary antibodies were incubated at room temperature for 30 minutes. |
| Instrument | BD FACSCalibur (Becton Dickinson) |
| Software | BD CellQuest Pro v4.0.2 (Acquisition)<br>FlowJo v10 (Analysis) |
| Cell population abundance | The major endocrine cell populations were in high prevalence in this study. Cell population abundances ranged from 5% to >50% depending on the time of maturation within the experiment. |
| Gating strategy | Cells were gated with FSC and SSC to remove small interfering cellular debris. Positive and negative gating was determined through negatively stained cells within the population and non-stained controls. |

☒ Tick this box to confirm that a figure exemplifying the gating strategy is provided in the Supplementary Information.
